# Mid-price prediction based on machine learning methods with technical and quantitative indicators

**DOI:** 10.1371/journal.pone.0234107

**Published:** 2020-06-12

**Authors:** Adamantios Ntakaris, Juho Kanniainen, Moncef Gabbouj, Alexandros Iosifidis

**Affiliations:** 1 Faculty of Information Technology and Communication Sciences, Tampere University, Tampere, Finland; 2 Department of Engineering, Electrical and Computer Engineering, Aarhus University, Aarhus, Denmark; Universidad Veracruzana, MEXICO

## Abstract

Stock price prediction is a challenging task, in which machine learning methods have recently been successfully used. In this paper, we extract over 270 hand-crafted features (factors) inspired by technical indicators and quantitative analysis and test their validity on short-term mid-price movement prediction for Nordic TotalView-ITCH stocks. The suggested feature list represents one of the most extensive studies in the field of financial feature engineering. We focus on a wrapper feature selection method using entropy, least-mean squares, and linear discriminant analysis. We also introduce a novel quantitative feature based on adaptive logistic regression for online learning. The proposed feature is consistently selected as the first feature among a large number of indicators used in this study. We further examine the best combinations of features using a high-frequency limit order book Nordic database. Our results suggest that sorting methods and classifiers can be used in such a way that one can reach the best classification performance with a combination of only a few advanced hand-crafted features.

## Introduction

The problem under consideration in this paper is the prediction of a stock’s mid-price movement (i.e., up, down, or stationary state) during high-frequency trading (HFT). At a given time instance, the mid-price of a stock is defined as the average of the best ask and bid prices. The mid-price is considered as vital information for market makers who continuously balance inventories as well as for traders who need to be able to correctly predict the direction of market movements. Moreover, the mid-price facilitates the process of monitoring the markets’ stability (i.e. spoofing identification). The concept of mid-price prediction can be described as follows: at a given time instance *t*, the state of the stock is encoded in a vector-based representation calculated using a multi-dimensional time series information from a short-term time window of length *T*. Given this representation, the direction of the mid-price is predicted at a horizon of Δ*t*.

**Fig 1:**
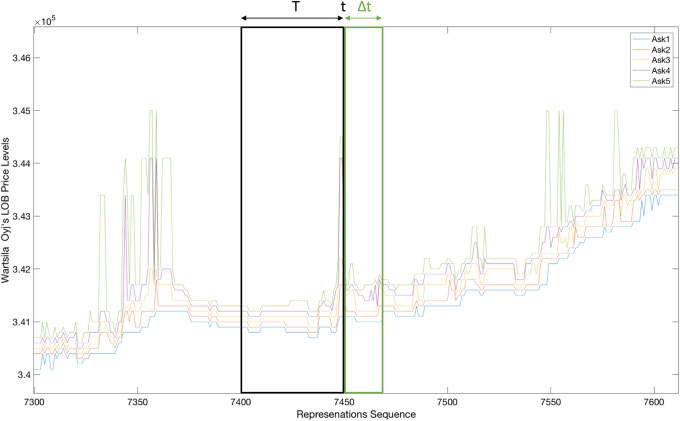
Mid-price prediction based on five limit order book ask price levels where *T* is the training feature extraction period and Δ*t* is the predicted horizon.

Over the past few years, several methods, such as those described in [1–6], and [7], have been proposed for analyzing stock market data. All of these methods follow the standard classification pipeline formed by two processing steps. Given a time instance during the trading process, the state of the market is described based on a (usually short) time window preceding the current instance. A set of hand-crafted features is selected to describe the dynamics of the market, leading to a vector representation. Based on such a representation, a classifier is then employed to predict the state of the market at a time instance within a prediction horizon, as illustrated in Fig 1.

The majority of the studies, as discussed in the Literature section, utilize a limited number of features without providing any motivation about their selection. In this paper, we employ a large number of the technical indicators, state-of-the-art limit order book (LOB) features, and quantitative indicators [8]. We further propose a novel quantitative feature that is selected first among several features for the task of mid-price movement prediction. The use of different hand-crafted features leads to encoding different properties of the financial time-series and excluding some of these features can result in failing to exploit the relevant information. The definition of a good set of features is directly connected to the performance of the subsequent analysis since any discarded information at this stage cannot be recovered later by the classifier.

A common approach to address this problem is the use of feature selection methods (e.g., [9, 10]) which can be performed in a wrapper fashion using various types of criteria for feature ranking. While the use of transformation-based dimensionality reduction techniques such as principal component analysis (PCA) or linear discriminant analysis can lead to a similar processing pipeline, in this paper, we are interested in defining the set of features that convey most of the information in the data. PCA is not considered in this study since it converts existing features to new ones which are not interpretable. That means that we will not be able to provide insight into which specific features are suitable for the task of mid-price movement prediction. The use of feature selection using unsupervised criteria, and in particular, the maximum entropy criterion, has been used in [11] and [12]. The motivation behind this approach is the fact that as the entropy of a feature increases (when it is calculated in a set of data), the data variance and, thus, the information it encodes, also increases. However, the combination of many high-entropy features in a vector-based representation does not necessarily lead to good classification performance. This is because different dimensions of the adopted data representation need to encode different information.

The main contribution of our work are three-fold. The first contribution is the use of an extensive list of technical indicators for high-frequency trading. The second contribution is a novel quantitative feature, named adaptive logistic regression feature, which was selected first among several feature selection metrics. The third contribution is an extensive evaluation of three feature sets (i.e., technical, quantitative, and LOB indicators) via the conversion of (i) entropy, (ii) linear discriminant analysis (LDA), and (iii) linear mean-square (LMS) as feature selection criteria. LMS, LDA, and radial basis function network (RBFN) are used as classifiers for the task of mid-price movement prediction task. Our findings suggest that the best performance is reached by using only a few (advanced) features derived from both quantitative and technical hand-crafted feature sets.

These different realizations (i.e., entropy, LMS, and LDA) of the feature selection method are applied to a wide pool of hand-crafted features, which are selected to cover both basic and advanced features from two different trading approaches (i.e., those focusing on technical and quantitative analyses). Technical analysis is based on the fact that price prediction can be achieved by monitoring price and volume charts, while quantitative analysis focuses on statistical models and parameter estimation. For the technical indicators, we calculate basic and advanced features accompanied by digital filters, while for the quantitative indicators, we primarily focus on time series analysis. The features and their respective descriptions are provided and used as input in twelve feature selection models (each corresponding to a different criterion and classifier combination) for the classification task. We present the best combinations of these two types of features and provide a comparison of the two trading styles of feature sets in terms of F1 performance. F1 score is a common test used to measure performance and is calculated as the harmonic mean of precision and recall. To the best of our knowledge, this is the first study to define which type of information needs to be used for high-frequency time series description and classification.

The remainder of the paper is organized as follows. We first provide a comprehensive literature review of the technical and quantitative features followed by the problem statement and data description. We then provide various realizations of the wrapper method adopted in our analysis, together with the empirical results. A detailed description of all features used in our experiments, as well as all ranking lists for each method, can be found in the Appendix section.

## Related work

Algorithmic trading uses computers, under specific rules, to rapidly perform accurate calculations based on statistical analysis. A trader using machine learning (ML) techniques can use several tools based on this analysis in order to select the best trading strategy. However, a number of challenges remain to be solved. First, how can one determine which indicators (i.e. features) are able to secure a profitable move? second, do past and present prices contain all the relevant information? Several authors utilized technical indicators and quantitative analysis for several tasks using only a limited set of these features. Hidden patterns extracted from past data as well as statistical models can provide relevant information to the ML trader.

Technical analysis (e.g., [13]) has traditionally received less academic scrutiny than quantitative analysis. Nevertheless, several studies employ technical indicators as the main mechanism for signal analysis and price prediction. In the sphere of HFT, authors in [14] utilize seven trading rule families as a measure of the impact of trading speed, while in [15] the authors provide only a few technical indicators for high-speed trading. In the current ML era, authors in [1] used six basic technical indicators as feature representations for a decision support system based on artificial neural networks (ANN). Only ten technical indicators are utilized in [16] as input features for several ML algorithms (i.e. ANN, Support Vector Machines, Random Forest, and Naive Bayes) to predict stock trends. However, one can also resort to quantitative analysis, which involves ML traders using complex mathematical and statistical indicators when making trading decisions. Quantitative finance is a broad field, including portfolio optimization (e.g., [17, 18]), asset pricing (e.g., [19, 20]), risk management (e.g., [21, 22]), and time series analysis (e.g., [23, 24]). In this work, we focus on time series analysis and use ideas from financial quantitative time series analysis that have been adopted to Machine Learning. For example, authors in [25] use support vector machines and decision trees via correlation analysis for stock market prediction. Another aspect of quantitative analysis is building trading strategies such as mean-reversion as tested in [26]). An additional aspect of quantitative analysis is the calculation of order book imbalance for order imbalance strategies. This idea is used as one of the features in a deep neural network in [4].

In the present work, we focus on extracted hand-crafted features based on technical and quantitative analysis. We show that a combination of features derived from these groups can improve the forecasting ability of the algorithms. A combined method is employed by [27] for asset returns predictability based on technical indicators and time series models. To the best of our knowledge this is the first attempt to compare these trading schools using several feature selection methods in a wrapper fashion in HFT.

## Problem formulation

HFT requires continuous analysis of market dynamics. One way to formulate these dynamics is by constructing a limit order book (LOB), as illustrated in Table 1. LOB is the cumulative order flow representing valid limit orders, that are not executed nor cancelled, which are listed in the so-called message list, as illustrated in Table 2. LOBs are multi-dimensional signals described by stochastic processes, and their dynamics are described as càdlàg functions (i.e., [2]). The functions are formulated for a specific limit order (i.e. an order with specific characteristics in terms of price and volume at a specific time t), as *t*), as: *order* = (*t*, *Price*_*t*_, *Volume*_*t*_) that becomes active at time *t* holds that: $order\in L\left(t\right),order\notin lim_{order^{\prime }↑order_{x}}L\left(order^{\prime }\right)$. Depending on how the LOB is constructed, we treat the new information according to event arrivals. The objective of our work is to predict the direction (i.e. up, down, or stationary) of the mid-price (i.e. (*p*_*a*_ + *p*_*b*_)/2, where *p*_*a*_ is the ask price and *p*_*b*_ is the bid price at the first level of LOB). The goal is to utilize informative features based on the order flow (i.e. message list or message book [MB]) and LOB, which will help the ML trader improve the accuracy of mid-price movement prediction.

**Table 1:** Limit order book example: Wartsila Oyj on 01 June 2010. LOB is divided in 10 levels, where each level consists of four columns. These four columns refer to the ask price and volume and the bid price and volume, respectively. The best level is Level 1. It contains the best Ask price which is the minimum price that a seller is willing to accept for a share of stock and the best Bid price which is the maximum price that a buyer is willing to pay for a share of stock. Next to Level 1 is Level 2 and so on, up to Level 10 with the same formation but with worst Ask and Bid prices.

			Level 1				Level 2				…
			Ask		Bid		Ask		Bid		
Timestamp	Mid-price	Spread	Price	Quantity	Price	Quantity	Price	Quantity	Price	Quantity	
1275386347944	126200	200	126300	300	126100	17	126400	4765	126000	2800	…
1275386347981	126200	200	126300	300	126100	17	126400	4765	126000	2800	…
1275386347981	126200	200	126300	300	126100	17	126400	4765	126000	2800	…
1275386348070	126050	100	126100	291	126000	2800	126200	300	125900	1120	…
1275386348070	126050	100	126100	291	126000	2800	126200	300	125900	1120	…
1275386348101	126050	100	126100	291	126000	2800	126200	300	125900	1120	…

**Table 2:** Message list example: Wartsila Oyj on 01 June 2010. This a typical message book which contains the raw trading information. Every message book row contains information regarding the trade arrival time, trade id, stock price, stock volume, event type and side of the trade.

Timestamp	Id	Price	Volume	Event	Side
1275377039033	1372349	341100	300	Submission	Bid
1275377039033	1372349	341100	300	Cancellation	Bid
1275377039037	1370659	343700	100	Submission	Ask
1275377039037	1370659	343700	100	Cancellation	Ask
1275377039037	1372352	341700	150	Submission	Bid
1275377039037	1372352	341700	150	Cancellation	Bid

## Feature pool

Limit Order Book (LOB) and Message Book (MB) are the main sources from which features are extracted. We provide a comprehensive list of features explored in the literature for technical and quantitative trading in Table 3. The description of the hand-crafted features, except the newly introduced feature, named Adaptive Logistic Regression feature, is provided in the Appendix where the description of the newly introduced feature, named Adaptive Logistic Regression feature, follows. The motivation for choosing the suggested list of features is based on an examination of all the basic and advanced features from technical analysis and comparisons with advanced statistical models, such as adaptive logistic regression for online learning. The present work has identified a gap in the existing literature concerning the performance of technical indicators and comparisons with quantitative models. This work sets the ground for future research since it provides insight into the features that are likely to achieve a high rank on the ordering list in terms of predictability power. To this end, we divide our feature sets into three main groups. The first group of features is extracted according to [28] and [29]. This group of features aims to capture the dynamics of the LOB. This is possible if we consider the actual raw LOB data and relative intensities of different look-back periods of the trade types (i.e. order placement, execution, and cancellation). The second group of features is based on technical analysis. The suggested list describes many of the existing technical indicators (basic and advanced). Technical indicators might help traders spot hidden trends and patterns in their time series. The third group of features is derived according to quantitative analysis, which is mainly based on statistical models; it can provide statistics that are hidden in the data. This can be verified by the ranking process, where the proposed advanced online feature (i.e. adaptive logistic regression) is ranked first in most of the feature selection metrics (i.e. four out of five feature lists). The suggested features are fully described in the Appendix; whereas, the proposed adaptive logistic regression feature is described next.

**Table 3:** Feature list for the three groups.

Feature Sets	Description
First group:	
Basic	n levels of LOB Data
Time-Insensitive	Spread & Mid-Price
	Price Differences
	Price & Volume Means
	Accumulated Differences
Time-Sensitive	Price & Volume Derivation
	Average Intensity per Type
	Relative Intensity Comparison
	Limit Activity Acceleration
Second group:	
Technical Analysis	
Accumulation Distribution Line	Awesome Oscillator
Accelerator Oscillator	Average Directional Index
Average Directional Movement Index Rating	Displaced Moving Average based on Williams Alligator Indicator
Absolute Price Oscillator	Aroon Indicator
Aroon Oscillator	Average True Range
Bollinger Bands	Ichimoku Clouds
Chande Momentum Oscillator	Chaikin Oscillator
Chandelier Exit	Center of Gravity Oscillator
Donchian Channels	Double Exponential Moving Average
Detrended Price Oscillator	Heikin-Ashi
Highest High and Lowest Low	Hull MA
Internal Bar Strength	Keltner Channels
Moving Average Convergence/Divergence Oscillator	Median Price
Momentum	Variable Moving Average
Normalized Average True Range	Percentage Price Oscillator
Rate of Change	Relative Strength Index
Parabolic Stop and Reverse	Standard Deviation
Stochastic Relative Strength Index	T3-Triple Exponential Moving Average
Triple Exponential Moving Average	Triangular Moving Average
Triple Exponential Average	True Strength Index
Ultimate Oscillator	Weighted Close
Williams %R	Zero-Lag Exponential Moving Average
Fractals	Linear Regression Line
Digital Filtering: Rational Transfer Function	Digital Filtering: Savitzky-Golay Filter
Digital Filtering: Zero-Phase Filter	Remove Offset and Detrend
Beta-like Calculation	
Third group:	
Quantitative Analysis	
	Autocorrelation
	Partial Correlation
	Cointegration based on Engle-Granger test
	Order Book Imbalance
	**Adaptive Logistic Regression**

### Proposed adaptive logistic regression feature

We introduce a novel logistic regression model that we use as a feature in our experimental protocol. The motivation for this model is the work done in [4] where the focal point is the local behavior of LOB levels. We extend this idea by doing online learning with an adaptive learning rate. The new feature operates under an online learning mechanism by taking into consideration the latest trading event of the 10-event message book block which means that the forecasting of the next mid-price move is updated according to the latest information flow. More specifically, we use the Hessian matrix as our adaptive rate. We also report the ratio of the logistic coefficients based on the relationship of the LOB levels close to the best LOB level and the ones which are deeper in the LOB. Since 0 ≤ *h*_*θ*_(*V*)≤1 and *V* are the stock volumes for the first best six levels of the LOB, we formulate the model as follows:

$$\begin{array}{c} h_{\theta }\left(V\right)=\frac{1}{1+e^{-\theta^{T}V}} \end{array}$$ (1)

be the logistic function (i.e. Hypothesis function) and $\theta^{T}V=\theta_{0}+\sum_{j=1}^{n}\theta_{j}V_{j}$. Parameter estimation is considered by calculating the parameter’s likelihood:

$$\begin{array}{c} L\left(\theta \right)=\prod_{i=1}^{m}{\left(h_{\theta }\left(V^{\left(i\right)}\right)\right)}^{y^{\left(i\right)}}{\left(1-h_{\theta }\left(V^{\left(i\right)}\right)\right)}^{1-y^{\left(i\right)}} \end{array}$$ (2)

for *m* training samples and the cost function, based on this probabilistic approach, is as follows:

$$\begin{array}{c} J\left(\theta \right)=\frac{1}{m}\sum_{i=1}^{m}[-y^{\left(i\right)}log\left(h_{\theta }\left(V^{\left(i\right)}\right)\right)-\left(1-y^{\left(i\right)}\right)log\left(1-h_{\theta }\left(V^{\left(i\right)}\right)\right)]. \end{array}$$ (3)

The next step is the process of choosing *θ*s for optimizing (i.e. minimizing) J(*θ*). To do so, we will use Newton’s update method:

$$\begin{array}{c} \theta^{\left(s+1\right)}=\theta^{\left(s\right)}-H^{-1}\nabla_{\theta }J, \end{array}$$ (4)

where the gradient is:

$$\begin{array}{c} \nabla_{\theta }J=\frac{1}{m}\sum_{1}^{m}\left(h_{\theta }\left(V^{\left(i\right)}\right)-y^{\left(i\right)}\right)V^{\left(i\right)} \end{array}$$ (5)

and the Hessian matrix is:

$$\begin{array}{c} H=\frac{1}{m}\sum_{i=1}^{m}[h_{\theta }\left(V^{\left(i\right)}\right)(1-h_{\theta }\left(V^{\left(i\right)}\right))V^{\left(i\right)}{\left(V^{\left(i\right)}\right)}^{T}] \end{array}$$ (6)

with $V^{\left(i\right)}{\left(V^{\left(i\right)}\right)}^{T}\in R^{\left(n+1)\times (n+1\right)}$ and *y*^(*i*)^ are the labels which are calculated as the differences of the best level’s ask (and bid) prices. The suggested labels describe a binary classification problem since we consider two states, one for change in the best ask price and another one for no change in the best ask price.

We perform the above calculation in an online manner. The online process considers the 9^th^ element of every 10 MB block multiplied by the *θ* coefficient first-order tensor to obtain the probabilistic behavior (we filter the obtained first-order tensor through the hypothesis function) of the 10^th^ event of the 10 MB block. The output is the feature representation expressed as a scalar (i.e. probability) of the bid and ask price separately.

## Wrapper method of feature selection

Feature selection is an area that focuses on applications with multidimensional datasets. A ML trader performs feature selection for three primary reasons: to reduce computational complexity, to improve performance, and to gain a better understanding of the underlying process. Feature selection, as a pre-processing method, can enhance classification power by adding features that contain information relevant to the task at hand. There are two metaheuristic feature selection methods: the wrapper method and the filter (i.e. transformation) -based method. We choose to perform classification based on the wrapper method since it considers the relationship among the features while filter methods do not.

Our wrapper approach consists of five different feature subset selection criteria based on two linear and one non-linear methods for evaluation with a general description in Algorithm 1 where: *l*_*opt*_ is the optimal feature list per criterion, *Feature*_*Select* is the name of the function algorithm, *X* is the input feature set, *labels* represent the movements of mid-price, *l* is the current feature list up to *D* feature dimensions, *N* is the sample size, *curr*_*X* is the current input data up to the current number *i* of the features together with the optimal ones up to *i*, and *best*_*d* is a vector that saves the best feature out of the entire list of features. More specifically, we convert entropy, LMS, and LDA as feature selection criteria. For the last two cases (i.e., LMS and LDA) we provide two different selection criteria as follows: i) for LMS the first metric follows the $L_{2}$-norm and the second metric is a statistical bias measure, and ii) for LDA the first metric is based on the ratio of the *within*-class scatter matrix while the second metric is derived according to the *between*-class scatter matrix. For classification evaluation we utilize LMS, LDA and a radial basis function network (i.e., [30]) as these were used in [29] and [31]. Our choice to apply these linear and non-linear classifiers is informed by the amount of data in our dataset (details will be provided in the following section). We measure classification performance according to accuracy, precision, recall, and F1 scores for every possible combination of the hand-crafted features by using LMS, LDA, and RBF ANN. These perfromance measures are defined as follows:

$$\begin{array}{c} Accuracy=\frac{TP+TN}{TP+TN+FP+FN} \end{array}$$ (7)

$$\begin{array}{c} Precision=\frac{TP}{TP+FP} \end{array}$$ (8)

$$\begin{array}{c} Recall=\frac{TP}{TP+FN} \end{array}$$ (9)

$$\begin{array}{c} F1=2\times \frac{Precision\times Recall}{Precision+Recall} \end{array}$$ (10)

where TP and TF represents the true positives and true negatives, respectively, of the mid-price prediction label compared with the ground truth, where FP and FN represents the false positives and false negatives, respectively.

**Algorithm 1** Wrapper-Based Feature Selection

1: **procedure** *l*_*opt*_ = *Feature*_*SelectX*, *labels*, *criterion*

2:  *l* = [1: *D*]

3:  *l*_*opt*_ = []

4:  *X*_*opt*_ = [], [*D*, *N*] = *size*(*X*)

5:  **for** *d* = 1: *D* **do**

6:   *crit*_*list* = []

7:   **for** *i* = 1: *D* − *d* + 1 **do**

8:    *curr*_*X* = [*X*_*opt*_;*X*(*i*;:)]

9:    *crit*_*list*[*i*] = *crit*(*curr*_*X*)

10:   **end for**

11:   [*best*_*d*, *best*_*crit*] = *opt*(*crit*_*list*)

12:   *l*_*opt*_[*d*] = *best*_*d*

13:   *l*[*best*_*d*] = []

14:   *X*_*opt*_ = [*X*_*opt*_;*X*(*best*_*d*;:)]

15:   *X*(*best*_*d*,:) = []

16:  **end for**

17: **end procedure**

### Feature sorting

**Fig 2:**
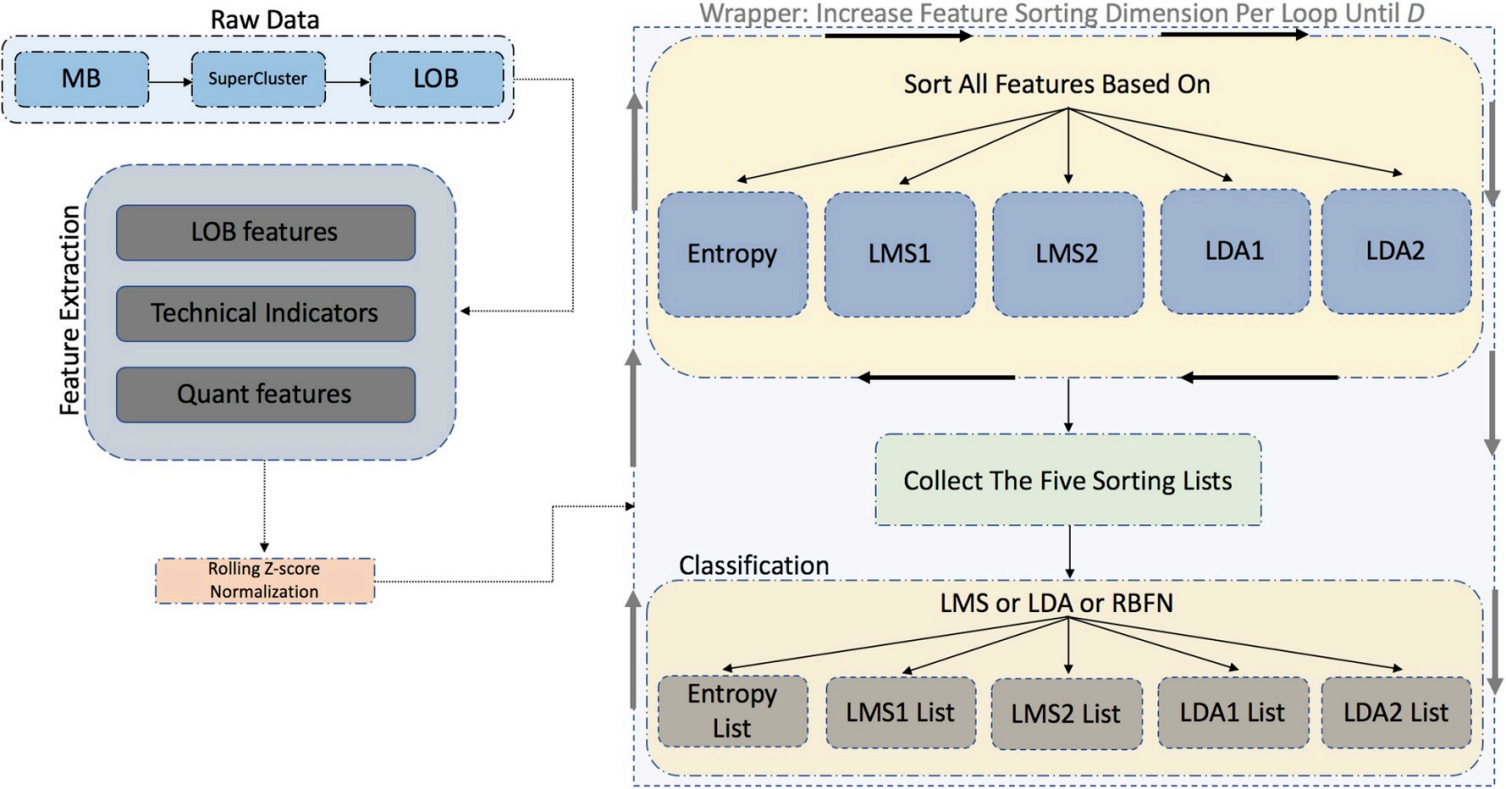
Wrapper method: Starting from the top left of the flow chart, we use MB and LOB data for feature extraction (i.e., LOB, technical and quantitative features). The next step is the z-score normalization of the suggested feature representations which are used as inputs to the wrapper protocol. The wrapper protocol is the main block of our experimental analysis. This main block is divided into two secondary blocks (i.e., yellow-colored blocks). The top yellow block acts as an incremental sorting method since it uses five different methods (i.e., entropy, LMS1, LMS2, LDA1, and LDA2) for feature evaluation. When the five sorted feature lists are ready then each sorted feature goes through a classifier (i.e., three different classifiers in our case based on RBFN, LDA, and LMS). This last part of the wrapper topology performs the classification task of the mid-price movement prediction.

We convert sample entropy, LMS, and LDA (for the latter two methods we use two different criteria for feature evaluation) into feature selection methods. A visual representation of the feature sorting method can be seen in Fig 2. It can be briefly described as follows: the process of feature sorting and classification is based on the wrapper method. From left to right: 1) Raw data is converted to LOB data via superclustering, 2) the feature extraction process follows (i.e., three feature sets are extracted), 3) then every feature set is normalized based on z-score in a rolling window, 4) Wrapper method: there are two main blocks in this process—the first block refers to sorting (i.e., five different sorting criteria based on entropy, LMS1, LMS2, LDA1, and LDA2) of the feature sets independently (i.e., LOB features, technical indicators, and quantitative indicators are sorted separately) and all together (i.e., the three feature sets are merged and sorted all together) and the second block refers to the incremental classification (i.e., we increase the dimension of each sorting list during classification by one feature in each loop and average the final F1 scores per sorting list) based on three classifiers (i.e., LMS, LDA, and RBFN).

#### Feature sorting with entropy

We employ entropy [32], which is a measure of signal complexity where the signal is the time series of the multidimensional two-mode tensor with dimensions $R^{p\times n}$, and where *p* is the number of features and n is the number of samples, as a measure of feature relevance. We calculate the bits of each feature in the feature set iteratively and report the order. We measure the entropy as follows:

$$\begin{array}{c} H\left(X\right)=-\sum_{i=1}^{p}p\left(x_{i}\right)\;log\;p\left(x_{i}\right), \end{array}$$ (11)

where *p*(*x*_*i*_) is the probability of the frequency per feature for the given data samples.

#### Feature sorting with least-mean-squares

We perform feature selection based on the least-mean square classification rate (LMS1) and $L_{2}$-norm (LMS2). LMS is a fitting method that aims to produce an approximation that minimizes the sum of squared differences between given data and predicted values. We use this approach to evaluate the relevance of our hand-crafted features. A hand-crafted feature evaluation is performed sequentially via LMS. More specifically, each of the features is evaluated based on the classification rate, the $L_{2}$-norm of the predicted labels, and the ground truth. The evaluation process is performed as follows:

$$\begin{array}{c} HW=T, \end{array}$$ (12)

where $H\in R^{p_{i}\times n}$ is the input data with feature dimension *p*_*i*_ which is calculated incrementally for the number of training samples *n*, $W\in R^{p_{i}\times \#cl}$ are the weighted coefficients for the number of features *p*_*i*_ of the number(#) of classes (i.e. up, down, and stationary labelling), and $T\in R^{\#cl\times n}$ represents the target labels of the training set. The weight coefficient matrix **W** is estimated via the following formula:

$$\begin{array}{c} W=H^{†}T, \end{array}$$ (13)

where **H**^†^ is the Moore-Penrose pseudoinverse matrix.

#### Feature sorting with linear discriminant analysis

Linear discriminant analysis (LDA) can be used for classification and dimensionality reduction. However, instead of performing these two tasks, we convert LDA into a feature selection algorithm. We measure feature selection performance based on two metrics. One is the classification rate (LDA1) and the other is based on the error term (LDA2), which we define as the ratio of the *within*-class scatter matrix and the *between*-class scatter matrix. The main objective of LDA is finding the projection matrix $W\in R^{m\times \#cl-1}$, where *m* is the sample dimension, and #*cl* is the number of classes, such that *Y* = *W*^*T*^ *X* maximizes the class separation. For the given sample set *X* = *X*_1_∪*X*_2_∪…∪*X*_#*cl*_, where $X_{k}={\left\{x_{1}^{k},...,x_{\ell_{k}}^{k}\right\}}_{k=1,...,\#cl}$ is the class-specific data subsample, we need to find **W** that maximizes the Fisher’s ratio:

$$\begin{array}{c} J\left(W\right)=\frac{trace(W^{T}S_{B}W)}{trace(W^{T}S_{W}W)}, \end{array}$$ (14)

where

$$\begin{array}{c} S_{B}=\sum_{i=1}^{\#cl}N_{i}\left(\mu_{i}-\mu \right){\left(\mu_{i}-\mu \right)}^{T} \end{array}$$ (15)

and

$$\begin{array}{c} S_{W}=\sum_{i=1}^{C}\sum_{x\in X_{i}}^{d}\left(x-\mu_{i}\right){\left(x-\mu_{i}\right)}^{T} \end{array}$$ (16)

are the *between*-class and *within*-class scatter matrices, respectively, with $\mu_{i}=\frac{1}{\ell_{k}}\sum_{k\in \#cl}^{}X_{k}$ and $\mu =\frac{1}{m}\sum_{k\in \#cl}^{}\ell_{k}X_{k}$. In a similar fashion, we compute the projected samples **y** with $\tilde{\mu_{i}}=\frac{1}{\ell_{k}}\sum_{k\in \#cl}^{}Y_{k}$ and $\tilde{\mu }=\frac{1}{m}\sum_{k\in \#cl}^{}\ell_{k}Y_{k}$, while the scatter matrices (i.e. *within* and *between* scatter matrices, respectively) are

$$\begin{array}{c} \tilde{S_{W}}=\sum_{i=1}^{\#cl}\sum_{k\in \#cl}\left(y-\tilde{\mu_{i}}\right){\left(y-\tilde{\mu_{i}}\right)}^{T} \end{array}$$ (17)

and

$$\begin{array}{c} \tilde{S_{B}}=\sum_{i=1}^{\#cl}\ell_{k}\left(\tilde{\mu_{i}}-\tilde{\mu }\right){\left(\tilde{\mu_{i}}-\tilde{\mu }\right)}^{T}. \end{array}$$ (18)

The above calculations constitute the basis for the two metrics that we use to evaluate 285 the hand-crafted features incrementally. The two evaluation metrics are based on the 286 classification rate and the ratio of the *within*-class and *between*-class scatter matrices of the projected space *Y*.

### Classification for feature selection

We perfrom classification evaluation based on three classifiers: LMS, LDA, and RBFN. The basic concept of the first two methods was discussed above while RBFN classifier is described in the following section.

#### RBFN classifier

We utilize a single-layer feedforward neural network (SLFN) as this suggested by [33]. A detailed description and the implementation of the method can be found in [29]. In order to train this model fast, we follow the procedures outlined in [34], and [35]. We use *K*-means clustering for *K* prototype vectors identification, which then are used as the network’s hidden layer weights. After determining the the network’s hidden layer weights $V\in R^{D\times K}$, the input data **x**_*i*_, *i* = 1, …, *N* are mapped to vectors $h_{i}\in R^{K}$ in the feature space determined by the network’s hidden layer outputs $R^{K}$. Then a radial basis function is used, i.e. **h**_*i*_ = *ϕ*_*RBF*_(**x**_*i*_), calculated in an element-wise manner, as follows:

$$\begin{array}{c} h_{ik}=\exp(\frac{∥x_{i}-v_{k}{∥}_{2}^{2}}{2\sigma^{2}}),\;\;k=1,\cdots ,K, \end{array}$$ (19)

where *σ* is a hyper-parameter denoting the spread of the RBF neuron and **v**_*k*_ corresponds to the *k*-th column of **V**.

The network’s output weights $W\in R^{K\times C}$ are determined by solving the following equation:

$$\begin{array}{c} W^{*}=\underset{W}{\arg min}\;\;∥W^{T}{H-T∥}_{F}^{2}+\lambda {∥W∥}_{F}^{2}, \end{array}$$ (20)

where **H** = [**h**_1_, …, **h**_*N*_] is a matrix formed by the network’s hidden layer outputs for the training data and **T** is a matrix formed by the network’s target vectors **t**_*i*_, *i* = 1, …, *N*. The network’s output weights are given by:

$$\begin{array}{c} W={\left(HH^{T}+\lambda I\right)}^{-1}HT^{T}. \end{array}$$ (21)

Then a new (test) sample $x\in R^{D}$ is mapped to its corresponding representations in spaces $R^{K}$ and $R^{C}$, i.e. **h** = *ϕ*_*RBF*_(**x**) and **o** = **W**^*T*^ **h**, respectively. Finally, the classification task is based on the maximal network output, i.e.:

$$\begin{array}{c} l_{x}=\underset{k}{\arg max}\;\;o_{k}. \end{array}$$ (22)

## Experimental results

In this section, we provide details regarding the conducted experiments. The goal of the experiments is to predict the mid-price state (i.e. up, down, and stationary) for ITCH feed data in millisecond resolution (ITCH is a type of direct data-feed protocol and the acronym according to Nasdaq carry no semantic meaning). Additional information regarding the dataset can be found in [29]). For the experimental protocol, we followed the setup in [29], which is based on the anchored cross-validation format. According to this format, we use the first day as training and the second day as testing for the first fold, whereas the second fold consists of the previous training and testing periods as a training set, and the next day is always used as the test set. Each of the training and testing sets contains the hand-crafted feature representations for all the five stocks from the FI-2010 dataset. Hence, we obtain a mode-three tensor of dimensions 273 × 458, 125. The first dimension is the number of features, whereas the second one is the number of sample events. At this point, we must specify that the process of hand-crafted feature extraction is conducted in the full length of the given information based on MB with 4,581,250 events. The motivation for taking separate blocks of messages of ten events is the time-invariant nature of the data. To keep the ML trader fully informed regarding MB blocks, we use features that convey this information by calculating, among others, price and volume averages, regression coefficients, risk factors, and online learning feedback indicators.

The results we present here are the mid-price predictions for the next 10^th^, 20^th^, and 30^th^ events (i.e. translated into MB events) or else one, two, and three next events after the current state translated into a feature representation setup. The prediction performance of these events is measured by the accuracy, precision, recall and F1 score, whereas F1 score is further emphasized, as it can only be affected in one direction by skewed distributions for unbalanced classes, as observed in our data. Performance metrics are calculated against mid-price labelling calculation of ground truth extraction. More specifically, we extract labels based on the percentage change of the smoothed mid-price with a span window of 9, for our supervised learning methods, computed as follows: $L_{1}=\frac{MP_{next}-MP_{curr}}{MP_{curr}}$, where *MP*_*curr*_ is the current mid-price, and *MP*_*next*_ is the next mid-price. The percentage change identification is thresholded by an empirically fixed value *γ* = 0.002 rolling z-score normalization is performed on the dataset to avoid look-ahead bias (look ahead bias refers to the process that future information is injected to the training set). The rolling window z-score normalization is based on the anchored cross-validation setup which means that the normalization of the training set is unaffected by any future information.

We report our results in Tables 4–8 for each possible combination of feature set, sorting and classification method used. RBFN classifier operates under the extreme learning machine model with a slight modification in the initialization process of weights calculation based on the K-means algorithm. The full description of this method can be found in [29]. We provide results based on the whole feature pool (see Table 4), the first feature pool according to [28] and [29] (see Table 5), based only on technical indicators (see Table 6) and the quantitative indicators (see Table 7). More specifically, for the first feature pool, we have 136 features, while for the second pool we have 82 features, and for the last pool we have 55 features; in total, we have 273 features.

**Table 4:** Results based on the total feature pool—273 features. Bold text highlights the best F1 performance per predicted horizon T. LMS classifier achieved the best F1 performance for every predicted horizon.

Sorting	Classifier	T	*Accuracy*	*Precision*	*Recall*	*F*1
Entropy	LMS	10	0.529 ± 0.059	0.447 ± 0.007	0.477 ± 0.013	0.440 ± 0.018
LMS1	LMS	10	0.540 ± 0.059	0.437 ± 0.007	0.456 ± 0.013	0.430 ± 0.018
LMS2	LMS	10	0.538 ± 0.052	0.447 ± 0.005	0.478 ± 0.013	**0.444** ± 0.011
LDA1	LDA	10	0.616 ± 0.048	0.408 ± 0.019	0.398 ± 0.011	0.397 ± 0.015
LDA2	LDA	10	0.543 ± 0.057	0.430 ± 0.010	0.455 ± 0.017	0.429 ± 0.018
LDA1	LMS	10	0.604 ± 0.068	0.468 ± 0.035	0.431 ± 0.042	0.408 ± 0.035
LDA2	LMS	10	0.522 ± 0.026	0.441 ± 0.020	0.473 ± 0.007	0.435 ± 0.007
Entropy	RBFN	10	0.474 ± 0.046	0.420 ± 0.031	0.445 ± 0.039	0.400 ± 0.039
LMS1	RBFN	10	0.600 ± 0.045	0.436 ± 0.019	0.425 ± 0.021	0.417 ± 0.019
LMS2	RBFN	10	0.537 ± 0.016	0.442 ± 0.011	0.470 ± 0.016	0.439 ± 0.012
LDA1	RBFN	10	0.585 ± 0.061	0.443 ± 0.018	0.438 ± 0.037	0.419 ± 0.026
LDA2	RBFN	10	0.528 ± 0.029	0.438 ± 0.020	0.467 ± 0.010	0.434 ± 0.017
Entropy	LMS	20	0.503 ± 0.049	0.469 ± 0.008	0.482 ± 0.014	**0.462** ± 0.017
LMS1	LMS	20	0.503 ± 0.049	0.470 ± 0.008	0.482 ± 0.014	**0.462** ± 0.017
LMS2	LMS	20	0.503 ± 0.049	0.469 ± 0.008	0.481 ± 0.014	**0.462** ± 0.018
LDA1	LDA	20	0.478 ± 0.060	0.400 ± 0.038	0.404 ± 0.041	0.393 ± 0.018
LDA2	LDA	20	0.505 ± 0.046	0.452 ± 0.009	0.461 ± 0.012	0.450 ± 0.012
LDA1	LMS	20	0.530 ± 0.032	0.457 ± 0.024	0.426 ± 0.048	0.401 ± 0.048
LDA2	LMS	20	0.499 ± 0.019	0.462 ± 0.019	0.476 ± 0.007	0.457 ± 0.015
Entropy	RBFN	20	0.464 ± 0.038	0.436 ± 0.033	0.448 ± 0.035	0.425 ± 0.036
LMS1	RBFN	20	0.519 ± 0.023	0.430 ± 0.016	0.417 ± 0.022	0.412 ± 0.027
LMS2	RBFN	20	0.508 ± 0.010	0.456 ± 0.015	0.466 ± 0.018	0.454 ± 0.017
LDA1	RBFN	20	0.523 ± 0.025	0.441 ± 0.024	0.429 ± 0.041	0.416 ± 0.046
LDA2	RBFN	20	0.502 ± 0.018	0.454 ± 0.019	0.465 ± 0.009	0.452 ± 0.015
Entropy	LMS	30	0.503 ± 0.042	0.475 ± 0.013	0.484 ± 0.014	0.470 ± 0.019
LMS1	LMS	30	0.503 ± 0.042	0.475 ± 0.013	0.484 ± 0.014	0.470 ± 0.019
LMS2	LMS	30	0.503 ± 0.043	0.474 ± 0.012	0.482 ± 0.014	0.461 ± 0.019
LDA1	LDA	30	0.464 ± 0.048	0.414 ± 0.025	0.420 ± 0.027	0.403 ± 0.018
LDA2	LDA	30	0.500 ± 0.043	0.457 ± 0.012	0.464 ± 0.013	0.455 ± 0.014
LDA1	LMS	30	0.489 ± 0.018	0.451 ± 0.030	0.429 ± 0.051	0.405 ± 0.072
LDA2	LMS	30	0.496 ± 0.016	0.476 ± 0.018	0.479 ± 0.009	**0.472** ± 0.015
Entropy	RBFN	30	0.464 ± 0.035	0.446 ± 0.035	0.449 ± 0.034	0.440 ± 0.037
LMS1	RBFN	30	0.471 ± 0.018	0.425 ± 0.018	0.414 ± 0.020	0.409 ± 0.026
LMS2	RBFN	30	0.494 ± 0.014	0.464 ± 0.021	0.466 ± 0.021	0.461 ± 0.024
LDA1	RBFN	30	0.481 ± 0.022	0.438 ± 0.034	0.428 ± 0.045	0.415 ± 0.057
LDA2	RBFN	30	0.493 ± 0.017	0.465 ± 0.018	0.467 ± 0.010	0.463 ± 0.016

**Fig 3:**
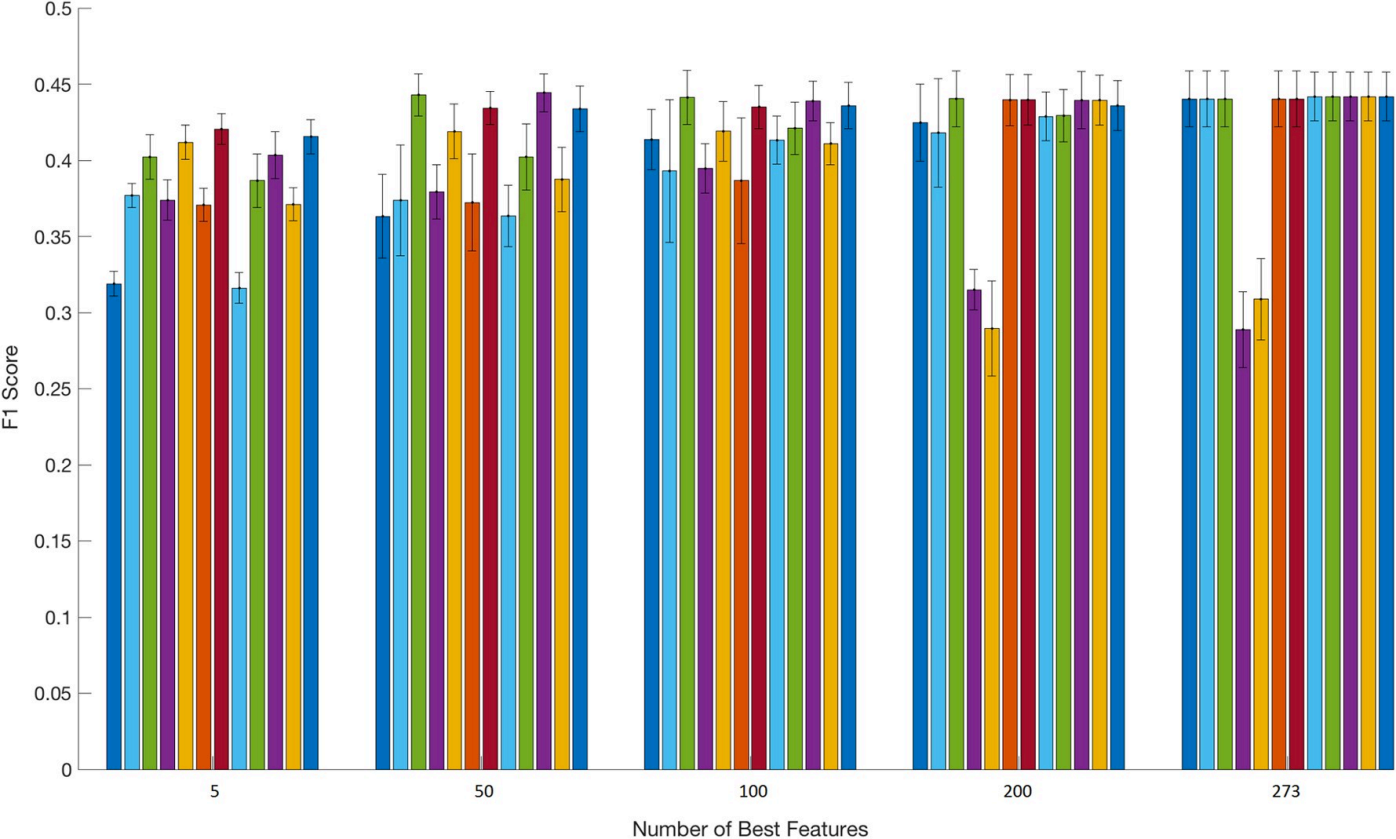
Bar plots for the F1 scores of the 12 different experimental models. This bar plot shows that an extensive feature selection mechanism is vital for a trader to identify the top candidates/indicators that can boost the classification performance. Several classifiers combined with a limited number of sorted hand-crafted features reached their top performance while some other classifiers reported lower F1 performance with more (sorted) features.

**Fig 4:**
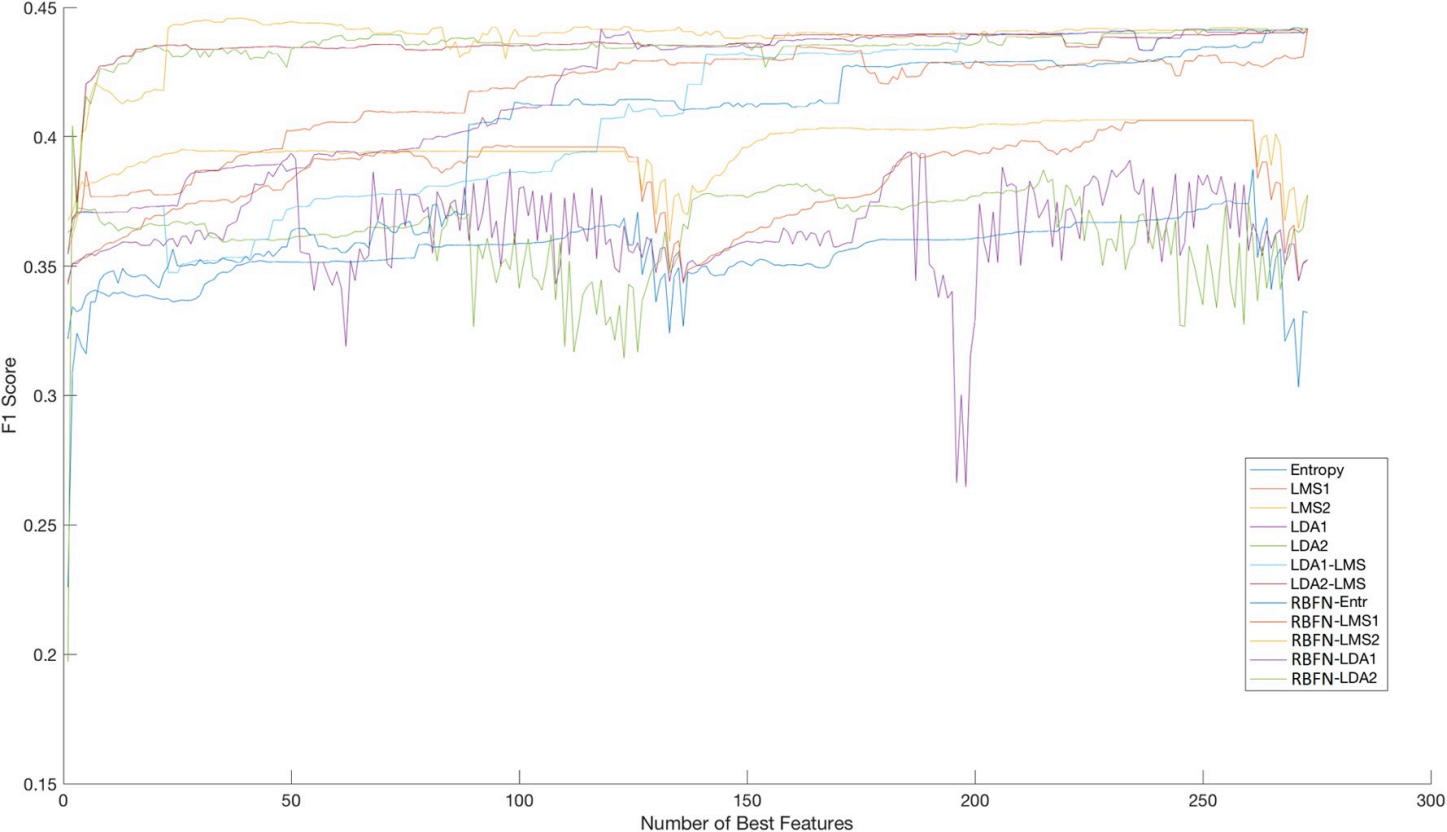
F1 performance per number of best features sequence for 10 events. This graph displays the same information as the bar plot above but also provides every model’s performance for every possible number of the sorted hand-crafted features.

The number of top features that used in the above methods is different in each case and can be monitored in Figs 3 and 4. We should point out that we tested all the possible combinations for the five sorting methods and the three classifiers (i.e., 15 different cases) but we report only results that exhibit some variations. For instance, in Tables 5–7 we report results for entropy as sorting method and LMS together with RBFN as classifiers but not with LDA (as a classifier) since the last method reports similar results. We focus on F1 score and particularly on F1-macro (i.e., F1-macro = $\frac{1}{C}\sum_{k\in C}F1_{k}$, with *C* as the number of classes for the 9-fold experimental protocol) results, based on the total feature pool, for the five sorting lists classified per LMS, LDA, and RBFN for the next *T* = 10^th^, 20^th^, and 30^th^ events, respectively, as the predicted horizon. Again, as the number of top features used in the above methods is different in each case (as seen in Fig 3), it can be briefly described as follows: Bar plots with variance presents the average (i.e. average F1 performance for the 9-fold protocol for all the features) F1 score of the 12 different models for the cases of 5, 50, 100, 200, and 273 number of top features. The order of the models from the left to the right column is:

1. feature list sorted based on entropy and classified based on LMS,
2. feature list sorted based on LMS1 and classified based on LMS,
3. feature list sorted based on LMS2 and classified based on LMS,
4. feature list sorted based on LDA1 and classified based on LDA,
5. feature list sorted based on LDA2 and classified based on LDA,
6. feature list sorted based on LDA1 and classified based on LMS,
7. feature list sorted based on LDA2 and classified based on LMS,
8. feature list sorted based on LDA2 and classified based on LMS,
9. feature list sorted based on entropy and classified based on RBFN,
10. feature list sorted based on LMS2 and classified based on RBFN,
11. feature list sorted based on LDA1 and classified based on RBFN, and
12. feature list sorted based on LDA2 and classified based on RBFN.

There is a dual interpretation of the suggested feature lists and wrapper method results. Regarding the feature lists, we have five different feature sorting methods starting from entropy, to LMS1 and LMS2 and continue to LDA1 and LDA2. More specifically, results based on the entropy sorting method reveal that the top 20 features almost entirely come from technical indicators (i.e. only 1 out of 20 comes from the first basic group), while the first 100 top ranked features include 36 quant features, 48 technical features, and 16 features from the first basic group.

For the LMS case, we present two sorting lists where we use two different criteria for the final feature selection. In the LMS1 case, the top 20 features are derived mainly from quantitative analysis (11 out of 20), 7 features come from the first basic group, and only 2 from the technical group of features. The top (best) feature is the proposed advanced feature based on the logistic regression model for online learning. For the same method, the first 100 best features include 25 quant features, 18 technical features, and the remaining 57 features come from the first basic group. In the LMS2 case, the first top 20 features include 7 quant, 9 technical, and only 4 features from the first basic group. LMS2 also selects the advanced feature based on the logistic regression model for online learning as its first top feature.

The last method that we use as the basis for the feature selection process is based on LDA. Similarly, we use two different criteria as a measure for the selection process. In LDA1, the first top 20 features include 10 quant, 3 technical, and 7 from the first basic group. The first top 100 features include 19 quant, 20 technical, and the remaining 61 features come from the first basic group. Again, the first top feature is the proposed advanced feature based on the logistic regression model for online learning. The last feature selection model, LDA2, selects 6 features from the quant pool, 6 from the technical pool, and 8 from the first basic group. LDA2 selects as the first top 100 features, 24 quant, 27 technical, and 49 features from the first basic group. The top 10 are listed for each of the 5 sorting methods in Table 9.

The second interpretation of our findings is the performance of the 12 different classifiers (based on LMS, LDA, and RBFN) used to measure, in terms of F1 score, the predictability of the mid-price movement. Fig 3 provides an overview of the F1 score performance in terms of best feature numbers and classifiers. We can divide these twelve models (pairs based on the sorting and classification method) into three groups according to their response in terms of information flow. The first group, where LMS2-LMS, LDA2-LMS, LMS2-RBFN, and LDA2-RBFN belong, reach their plateau very early in the incremental process of adding less informative features. These models were able to reach (close to) their maximum F1 score performance with approximately 5 top features, which means that the dimensionality of the input matrix to the classification model is quite small. The second group of models, Entropy-LMS, LMS1-LMS, LDA1-LMS, Entropy-RBFN, LMS1-RBFN, and LDA1-RBFN, had a slower reaction in the process of reaching their top F1 score performance. The last group of models, LDA1-LDA and LDA2-LDA, reached their best performance (which is not higher than that of the other models) very early in the process with only five features.

The conducted experiments show that this quantitative analysis can provide significant trading information; however, the results improve when technical features are incorporated in the feature set. All top listed features include the logistic regression model based feature. This shows that more advanced quantitative features may provide the ML trader with vital information regarding metrics prediction. One implication of the proposed experimental protocol is that the development of advanced hand-crafted features as part of a wrapper framework requires from the ML trader to compare and combine several sets until a target level is reached.

## Conclusion

In this paper, we proposed extracted hand-crafted features inspired by technical and quantitative analysis and tested their validity on the mid-price movement prediction task. We introduced a novel quantitative feature based on adaptive logistic regression for online learning and used a wrapper feature selection method by utilizing entropy, least-mean squares, and linear discriminant analysis to guide feature selection combined with linear and non-linear classifiers. This work is the first attempt of this extent to develop a framework in information edge discovery via informative hand-crafted features. Therefore, we provided the description of three sets of hand-crafted features suitable for high-frequency trading (HFT) by considering each 10-message book block as a separate trading unit (i.e., trading days).

We evaluated our experimental framework on five ITCH feed data stocks from the Nordic stock market. The dataset contained over 4.5 million events which were incorporated into the hand-crafted features. The results suggest that sorting methods and classifiers can be combined in such a way that market makers and traders can reach, with only a few informative features, top performance levels. Furthermore, the proposed advanced quantitative feature based on logistic regression for online learning has most of the time been selected as the top feature by the sorting methods. This is a strong indication for future research on developing more advanced features combined with more sophisticated feature selection methods. Classification performance can be easily improved by using more advanced classifiers such as convolutional neural networks and recurrent neural networks. Our work opens avenues for other applications as well. For instance, the same type of analysis is suitable for exchange rates and bitcoin time series analysis. As part of our future work, we also intend to test our experimental protocol on a longer trading periods.

## Appendix

### 1 Feature pool

#### First group of features

This set of features is based on the work in [28] and [29] and is divided into three groups: basic, time-insensitive, and time-sensitive features. These are fundamental features since they reflect the raw data directly without any statistical analysis or interpolation. We calculated them as follows:

**Basic**

- $n\;\text{LOB}\;\text{Levels}={\left\{P_{i}^{ask},V_{i}^{ask},P_{i}^{bid},V_{i}^{bid}\right\}}_{i=1}^{n}$

which represents the raw data of the 10 levels of our LOB, where $P_{i}^{ask}$, $V_{i}^{ask}$, $P_{i}^{bid}$, and $V_{i}^{bid}$ are the Prices and Volumes for the ask and bid sides for every LOB level *i*, respectively.

**Time-Insensitive**

- $\text{Spread}\&\text{Mid-Price}={\left\{\left(P_{i}^{ask}-P_{i}^{bid}\right),\left(P_{i}^{ask}+P_{i}^{bid}\right)/2\right\}}_{i=1}^{n}$
- $\text{Price}\;\text{Differences}=\{P_{n}^{ask}-P_{1}^{ask},P_{1}^{bid}-P_{n}^{bid},|P_{i+1}^{ask}-P_{i}^{ask}\left|,\right|P_{i+1}^{bid}-P_{i}^{bid}{\left|\right\}}_{i+1}^{n}$
- $\text{Price}\&\text{Volume}\;\text{Means}=\{\frac{1}{n}\sum_{i=1}^{n}P_{i}^{ask},\frac{1}{n}\sum_{i=1}^{n}P_{i}^{bid},\frac{1}{n}\sum_{i=1}^{n}V_{i}^{ask},\frac{1}{n}\sum_{i=1}^{n}V_{i}^{bid}\}$
- $\text{Accumulated}\;\text{Differences}=\{\sum_{i=1}^{n}\left(P_{i}^{ask}-P_{i}^{bid}\right),\sum_{i=1}^{n}\left(V_{i}^{ask}-V_{i}^{bid}\right)\}$.

**Time-Sensitive**

- $\text{Price}\&\text{Volume}\;\text{Derivations}={\left\{dP_{i}^{ask}/dt,dP_{i}^{bid}/dt,dV_{i}^{ask}/dt,dV_{i}^{bid}/dt\right\}}_{i=1}^{n}$
- $\text{Average}\;\text{Intensity}\;\text{Per}\;\text{Type}=\{\lambda_{\Delta t}^{1},\lambda_{\Delta t}^{2},\lambda_{\Delta t}^{3},\lambda_{\Delta t}^{4},\lambda_{\Delta t}^{5},\lambda_{\Delta t}^{6}\}$
- $\text{Relative}\;\text{Intensity}\;\text{Comparison}=\{1_{\lambda_{\Delta_{t}}^{1}>\lambda_{\Delta_{T}}^{1}},1_{\lambda_{\Delta_{t}}^{2}>\lambda_{\Delta_{T}}^{2}},1_{\lambda_{\Delta_{t}}^{3}>\lambda_{\Delta_{T}}^{3}},1_{\lambda_{\Delta_{t}}^{4}>\lambda_{\Delta_{T}}^{4}},1_{\lambda_{\Delta_{t}}^{5}>\lambda_{\Delta_{T}}^{5}},1_{\lambda_{\Delta_{t}}^{6}>\lambda_{\Delta_{T}}^{6}}\}$
- Limit Activity Acceleration = {*d*λ^1^/*dt*, *d*λ^2^/*dt*, *d*λ^3^/*dt*, *d*λ^4^/*dt*, *d*λ^5^/*dt*, *d*λ^6^/*dt*}.

where λ denotes the average short or longer-term intensity per type and horizon.

#### Technical analysis

Technical analysis is based mainly on the idea that past data provides all the relevant information for trading prediction. The prediction, based on technical analysis, takes place according to open-close-high and low prices in day-to-day trading. We adjust this idea to the HFT ML problem for every 10-MB block of events. More specifically, we consider every 10-MB block as a ‘trading’ day (i.e. with *t* as the current 10-MB block and *t*-1 the previous 10-MB block), and we extract features according to this formation as follows:

*Accumulation Distribution Line*. Accumulation Distribution Line (ADL) [36] is a volume-based indicator for measuring supply and demand and is a three-step process:

- *MoneyFlowMultiplier* = [(*C*_*t*_ − *L*_*t*_) − (*H*_*t*_ − *C*_*t*_)]/(*H*_*t*_ − *L*_*t*_)
- *MoneyFlowVolume*_*t*_ = *MoneyFlowMultiplier* x *BlockPeriodVolume*
- *ADL* = *ADL*_*t*−1_ + *MoneyFlowVolume*_*t*_

with *C*_*t*_, *L*_*t*_, and *H*_*t*_ being the closing, lowest, and highest current 10-MB block prices, respectively, and *BlockPeriodVolume*, *ADL*_*t*−1_, and *MoneyFlowVolume*_*t*_ are the total amounts of 10-MB block volume density, the previous ADL price, and the current *MoneyFlowVolume*_*t*_, respectively.

*Awesome oscillator*. An awesome oscillator (AO) [37] is used to capture market momentum. Here, we adjust the trading rules according to the previous block horizon investigation to 5 and 34 previous 10-MB blocks as follows:

- AO = *SMA*_5_((*H*_*t*_ + *L*_*t*_)/2) − *SMA*_34_((*H*_*t*_ + *L*_*t*_)/2)

where *SMA*_5_ and *SMA*_34_ are the simple moving averages of the previous 5 and 34 previous blocks, respectively.

*Accelerator oscillator*. An accelerator oscillator [37] is another market momentum indicator derived from AO. It is calculated as follows:

- *AC* = *AO* − *SMA*_5_(*AO*)

*Average directional index*. An average directional index (ADX) indicator [38] has been developed to identify the strength of a current trend. The ADX is calculated as follows:

- *TR* = *max*(*H*_*t*_ − *L*_*t*_, |*H*_*t*_ − *CL*_*t*−1_|, |*L*_*t*_ − *CL*_*t*−1_|)
- + *DM* = *H*_*t*_ − *H*_*t*−1_
−*DM* = *L*_*t*_ − *L*_*t*−1_
- *TR*_14_ = *TR*_*t*−1_ − (*TR*_*t*−1_/14) + *TR*
- + *DM*_14_ = (+ *DL*_*t*−14_) − ((+ *DL*_*t*−14_)/14) + (+ *DM*)
−*DM*_14_ = (−*DL*_*t*−14_) − ((−*DL*_*t*−14_)/14) + (−*DM*)
- + *DI*_14_ = 100 × ((+ *D*_14_)/(+ *TR*_14_))
−*DI*_14_ = 100 × ((−*D*_14_)/(−*TR*_14_))
- $DI_{diff_{14}}=\left|\left(+D_{14}\right)-\left(-D_{14}\right)\right|$
- $DI_{sum_{14}}=\left|\left(+D_{14}\right)+\left(-D_{14}\right)\right|$
- $DX=100\times (\left(DI_{diff_{14}}\right)/\left(DI_{sum_{14}}\right))$
- *ADX* = (*ADX*_*t*−1_ × 13) + *DX*)/14

where TR = true range, *H*_*t*_ = the current 10-block’s highest MB price, *L*_*t*_ = the current 10-block’s lowest MB price, *CL*_*t*_ = the previous 10-block’s closing MB price, + *DM* = positive Directional Movement (DM), −*DM* = negative DM, *TR*_14_ = TR based on the previous 14-blocks, *TR*_*t*−1_ = the previous TR price, + *DM*_14_ = DM based on the previous 14 + *DM* blocks, −*DM*_14_ = DM based on the previous 14 −*DM* blocks, + *DM*_*t*−14_ = +DM of the previous 14 + *DM* blocks, $DI_{diff_{14}}$ = is the directional indicator (DI) of the difference between + *DM*_14_ and −*DM*_14_, $DI_{sum_{14}}$ = DI of the sum between + *DM*_14_ and −*DM*_14_, *DX* = directional movement index and *ADX*_*t*−1_ = the previous average directional index.

*Average directional movement index rating*. An average directional movement index rating (ADXR) evaluates the momentum change of ADX, and it is calculated as the average of the current and previous price of ADX:

- *ADXR* = (*ADX*+ *ADX*_*t*−1_)/2

*Displaced moving average based on williams alligator indicator*. A displaced moving average [39] is the basis for building a trading signal named Alligator. In practice, this is a combination of three moving averages (MA). We adjust this idea as follows:

- *Alligator*_*Jaw*_ = *SMA*_13_((*H*_*t*_ + *L*_*t*_)/2)
- *Alligator*_*Teeth*_ = *SMA*_8_((*H*_*t*_ + *L*_*t*_)/2)
- *Alligator*_*Lips*_ = *SMA*_5_((*H*_*t*_ + *L*_*t*_)/2)

where *SMA*_13_((*H*_*t*_ + *L*_*t*_)/2), *SMA*_8_((*H*_*t*_ + *L*_*t*_)/2), and *SMA*_5_((*H*_*t*_ + *L*_*t*_)/2) are the simple moving averages based on the previous 13, 8, and 5 average highest and lowest block prices, respectively.

*Absolute price oscillator*. An absolute price oscillator (APO) belongs to the family of price oscillators. It is a comparison between fast and slow exponential moving averages and is calculated as follows:

- *M*_*t*_ = (*H*_*t*_ + *L*_*t*_)/2
- APO = *EMA*_5_(*M*_*t*_) − *EMA*_13_(*M*_*t*_)

where *EMA*_5_(*M*_*t*_) and *EMA*_13_(*M*_*t*_) are the exponential moving averages of range 5 and 13 periods, respectively, for the average of high and low prices of the current 10-MB block.

*Aroon indicator*. An Aroon indicator [40] is used as a measure of trend identification of an underlying asset. More specifically, the indicator has two main bodies: the uptrend and downtrend calculation. We calculate the Aroon indicator based on the previous twenty 10-MB blocks for the highest-high and lowest-low prices, respectively, as follows:

- $Arron_{Up}=(20-H_{high_{20}}/20)\times 100$
- $Arron_{Down}=(20-L_{low_{20}}/20)\times 100$

where $H_{high_{20}}$ and $L_{low_{20}}$ are the highest-high and lowest-low 20 previous 10-MB block prices, respectively.

*Aroon oscillator*. An Aroon oscillator is the difference between *Aroon*_*Up*_ and *Aroon*_*Down*_ indicators, which makes their comparison easier:

- Arron Oscillator = *Aroon*_*Up*_—*Aroon*_*Down*_

*Average true range*. Average true range (ATR) [41] is a technical indicator which measures the degree of variability in the market and is calculated as follows:

- ATR = (*ATR*_*t*−1_ × (*N* − 1)+ *TR*)/*N*

Here we use N = 14, where N is the number of the previous 10-TR values, and *ATR*_*t*−1_ is the previous ATR 10-MB block price.

*Bollinger bands*. Bollinger bands [42] are volatility bands which focus on the price edges of the created envelope (middle, upper, and lower band) and can be calculated as follows:

- *BB*_*middle*_ = *SMA*_20_(*CL*)
- $BB_{upper}=SMA_{20}\left(CL\right)+BB_{std_{20}}\times 2$
- $BB_{lower}=SMA_{20}\left(CL\right)-BB_{std_{20}}\times 2$

where *BB*_*middle*_, *BB*_*upper*_, and *BB*_*lower*_ represent the middle, upper, and lower Bollinger bands, *SMA*_20_(*CL*) represents the simple moving average of the previous twenty 10-block closing prices, and $BB_{std_{20}}$ represents the standard deviation of the last twenty 10-MB blocks.

*Ichimoku clouds*. Ichimoku clouds [43] are ‘one glance equilibrium charts,’ which means that the trader can easily identify a good trading signal and is possible since this type of indicator contains dense information (i.e. momentum and trend direction). Five modules are used in an indicator’s calculation:

- Conversion Line (*Tenkan*–*sen*) = (*H*_9_ + *L*_9_)/2
- Base Line (*Kijun*–*sen*) = *H*_26_ + *L*_26_
- Leading Span A (*Senkou* *Span* *A*) = (Conversion Line + Base line)/2
- Leading Span B (*Senkou* *Span* *B*) = (*H*_52_ + *L*_52_)/2
- Lagging Span (*Chikou* *Span*) = *CL*_26_

where H, L, and CL denote the highest, lowest, and closing prices of the 10-MB raw data, respectively, where subscripts 9, 26, and 52 denote the past horizon of our trading rules, respectively.

*Chande momentum oscillator*. A Chande momentum oscillator (CMO) [40] belongs to the family of technical momentum oscillators and can monitor overbought and oversold situations. There are two modules in the calculation process:

- $S_{u}=\sum_{i=1}^{19}CL_{i}\times 1_{CL_{t}>CL_{t-19}}$
- $S_{d}=\sum_{i=1}^{19}CL_{i}\times 1_{CL_{t}<CL_{t-19}}$
- CMO = 100 × (*S*_*u*_—*S*_*d*_)/(*S*_*u*_ + *S*_*d*_)

where *CL*_*i*_ is the 10-block’s closing price with *i* = 1, and *CL*_*t*_ and *CL*_*t*−19_ are the current block’s closing price and the 19 previous blocks’ closing prices, respectively.

*Chaikin oscillator*. The main purpose of a Chaikin oscillator [44] is to measure the momentum of the accumulation distribution line as follows:

- *MFM* = (*CL*_*t*_ − *L*_*t*_) − (*H*_*t*_ − *CL*_*t*_)]/(*H*_*t*_ − *L*_*t*_)
- $MFV=MFM\times \sum_{j=1}^{10}V_{j}$
- *ADL* = *ADL*_*t*−1_ + *MFM*
- Chaikin Oscillator = *EMA*_3_(*ADL*)—*EMA*_10_(*ADL*)

where *MFM* and *MFV* stand for *Money Flow Multiplier* and *Money Flow Volume*, respectively, V is the volume of each of the trading events in the 10-block MB, and *EMA*_3_(*ADL*) and *EMA*_10_(*ADL*) are the exponential moving average for the past 3 and 10 10-MB blocks, respectively.

*Chandelier exit*. A Chandelier exit [45] is part of the trailing stop strategies based on the volatility measured by the ATR indicator. It is separated based on the number of ATRs that are below the 22-period high (long) or above the 22-period low (short) and is calculated as follows:

- *Chandelier*_*Long*_ = *H*_22_ − *ATR*_22_ × 3
- *Chandelier*_*Short*_ = *L*_22_ + *ATR*_22_ × 3

where *H*_22_ and *L*_22_ denote the highest and lowest prices for a period of 22 10-MB blocks, and *ATR*_22_ are the ATR values for the 22 previous 10-MB blocks.

*Center of gravity oscillator*. The center of gravity oscillator (COG) [46] is a comparison of current prices against older prices within a specific time window and is calculated as follows:

- *M*_*t*_ = (*H*_*t*_ + *L*_*t*_)/2
- COG = −(*M*_*t*_ + *r* × *M*_*t*−1_)/(*M*_*t*_ + *M*_*t*−1_)

where *M*_*t*_ is the current mid-price of the highest and lowest prices of each of the 10-MB blocks, and r is a weight that increases according to the number of the previous *M*_*t*−1_ prices.

*Donchian channels*. The Donchian channel (DC) [47] is an indicator which bands the signal and notifies the ML trader of a price breakout. There are three modules in the calculation process:

- $DC_{upper}=H_{high_{20}}$
- $DC_{lower}=L_{low_{20}}$
- $DC_{middle}=(H_{high_{20}}+L_{low_{20}})/2$

where $H_{high_{20}}$ and $L_{low_{20}}$ are the highest high and lowest low prices of the previous twenty 10-MB blocks.

*Double exponential moving average*. A double exponential moving average (DEMA) [48] provides a smoothed average and offers a diminished amount of delays as follows:

- *M*_*t*_ = (*H*_*t*_ + *L*_*t*_)/2
- *DEMA* = 2 × *EMA*_20_(*M*_*t*_) − *EMA*_20_(*EMA*_20_(*M*_*t*_))

where *EMA*_20_ is the exponential moving average of span 20 of the closing prices under the 10-MB block format.

*Detrended price oscillator*. A detrended price oscillator (DPO) is an indicator used for short-term and long-term signal identification. The DPO eliminates cycles that are longer than the MA horizon. On day-to-day trading, the closing prices are considered for the calculation, but here, we use the highest 10-MB block price as follows:

- $DPO=\left(H_{high_{10}}/\left(10+2\right)\right)-SMA_{10}\left(CL\right)$.

*Heikin-Ashi*. Heikin-Ashi [49] is a candlestick method and is described as a visual technique that eliminates irregularities:

- *Heikin*_*Close*_ = (*O*_*t*_ + *H*_*t*_ + *L*_*t*_ + *CL*_*t*_)/4
- *Heikin*_*Open*_ = (*O*_*t*−1_ + *CL*_*t*−1_)/2
- *Heikin*_*High*_ = *max*(*H*_*t*_, *O*_*t*−1_, *CL*_*t*−1_)
- *Heikin*_*Low*_ = *min*(*L*_*t*_, *O*_*t*−1_, *CL*_*t*−1_)

where *O*_*t*−1_ and *CL*_*t*−1_ are the open and close prices of the previous 10-MB block.

*Highest high and lowest low*. Highest high and lowest low creates an envelope of the trading signal for the last twenty 10-MB blocks:

- $Highest_{High}=H_{high_{20}}$
- $Lowest_{Low}=L_{low_{20}}$

*Hull MA*. A Hull moving average is a weighted moving average that reduces the smoothing lag effect by using the square root of the block period. It is calculated as follows:

- $HULL_{MA}=WMA_{\sqrt{10}}\left(AHL\right)\left(2\times WMA_{5}\left(AHL\right)-WMA_{10}\left(AHL\right)\right)$

where *WMA*_5_(*AHL*) and *WMA*_10_(*AHL*) denote the weighted moving average of the average high and low 10-MB block for periods 5 and 10, respectively.

*Internal bar strength*. Internal bar strength (IBS) [50] is based on the position of the day’s closing price in relation to the day’s range where we adjust this idea to the 10-MB block setup as follows:

- *IBS* = (*CL*_*t*_ − *L*_*t*_)/(*H*_*t*_ − *L*_*t*_).

*Keltner channels*. Keltner channels [51] are based on Bollinger bands. The main difference, for this volatility-based indicator, is that it uses ATR instead of standard deviation, as follows:

- $Middle_{Channle}=EMA_{20_{AHL}}$
- *Upper*_*Channel*_ = *Middle*_*Channel*_+ 2 × *ATR*_10_
- *Lower*_*Channel*_ = *Middle*_*Channel*_ − 2 × *ATR*_10_.

*Moving Average Convergence/Divergence Oscillator (MACD)*. A moving average convergence/divergence oscillator [52] is a measure of the convergence and divergence of two moving averages and is calculated as follows:

- *MACD* = *EMA*_12_(*AHL*) − *EMA*_26_(*AHL*)

where AHL is the average of high and low prices for 12 and 26 previous 10-MB blocks, respectively, with *EMA*_12_(*AHL*) and *EMA*_26_(*AHL*) as the exponential moving average of AHL of span 12 and 26, respectively.

*Median price*. Median price is an indicator which simplifies the price overview. We calculate this indicator based on the 10-MB block highest and lowest average prices:

- *Median*_*t*_ = (*H*_*t*_ + *L*_*t*_)/2

*Momentum*. A momentum (MOM) indicator measures the rate of change of the selected time series. In our case, we calculate it based on closing prices:

- *MOM* = *CL*_*t*_ − *CL*_*t*−1_.

*Variable moving average*. A variable moving average (VMA) [53] is a dynamic indicator which acts as a variable-length moving average with volatility-adaptation capabilities. We calculate VMA based on the efficiency ratio (ER) as follows:

- *Direction* = |*CL*_*t*_ − *CL*_*t*−3_|
- $Volatility=3\times \sum_{ii=1}^{3}\left|CL_{ii}-CL_{ii+1}\right|$
- *ER* = *Direction*/*Volatility*
- $VMA=\sum_{jj=1}^{3}\alpha \times ER_{jj}\times CL_{jj}$

where *α* = 2/(*N*+ 1), for N = 3 previous 10-MB blocks, is the adaptive parameter.

*Normalized average true range*. A normalized average true range (NATR) normalizes the average true range as follows:

- *NATR* = (*ATR*/*CL*_*t*_) × 100.

*Percentage price oscillator*. A percentage price oscillator (PPO) displays the convergence and divergence of two moving averages and focuses on the percentage change of the larger moving average, as follows:

- *MACD* = *EMA*_12_(*AHL*) − *EMA*_26_(*AHL*)
- *PPO* = (*MACD*/*EMA*_26_(*AHL*)) × 100.

*Rate of change*. Rate of change (ROC) measures the ascent or descent speed of the time series change:

- *ROC* = (*CL*_*t*_ − *CL*_*t*−12_/*CL*_*t*−12_) × 100.

*Relative strength index*. A relative strength index (RSI) [38] is a measure of the velocity and magnitude of directional time series movements and is calculated as follows:

- *CL*_*d*_ = *CL*_*t*_ − *CL*_*t*−1_
- $AG_{14}=\sum_{l=1}^{14}CL_{d_{l}}1_{CL_{d_{t}}>CL_{d_{t-1}}}$
- $AL_{14}=\sum_{l=1}^{14}CL_{d_{l}}1_{CL_{d_{t}}<CL_{d_{t-1}}}$
- *Relative*_*Strength*_ = *AG*_14_/*AL*_14_
- *RSI* = 100 − 100/(1+ *Relative*_*Strength*_)

where *AG*_14_ and *AL*_14_ denotes the average gain and loss of the last fourteen 10-MB blocks, respectively.

*Parabolic stop and reverse*. Parabolic SAR (PSAR) [41] is a trend following indicator which protects profits. There are two main modules for its calculation, the Rising SAR and the Falling SAR, and they are calculated as follows:

- Rising SAR
  - *AF* = *Incremental* *increase* *of* *a* *predefined* *step*
  - $EP=H_{High_{5}}$
  - *SAR* = *SAR*_*t*−1_ + *AF*_*t*−1_(*EP*_*t*−1_ − *SAR*_*t*−1_)
- Falling SAR
  - *AF* = *Incremental* *increase* *of* *a* *predefined* *step*
  - $EP=L_{Low_{5}}$
  - *SAR* = *SAR*_*t*−1_ − *AF*_*t*−1_(*EP*_*t*−1_ − *SAR*_*t*−1_)

where *AF* is the acceleration factor, and *EP* is the extreme point

*Standard deviation*. Standard deviation is a measure of volatility. We calculate this indicator based on the closing prices of every 10-MB block, as follows:

- *Deviation* = *CL*_*t*_ − *SMA*_10_(*CL*)
- $SASD=\sqrt{SMA_{10}\left(SVD\right)}$

where *SMA*_10_(*CL*) is the simple moving average of the last 10 closing 10-MB prices, *SASD* is the squared deviation of the SMA of the standard deviation (SVD) of the last 10 closing values of our 10-MB blocks.

*Stochastic relative strength index*. A stochastic relative strength index (Stoch RSI) [40] is a range-bound momentum oscillator which provides information for the RSI based on the closing prices in terms of high and low stock prices:

- $Stoch_{RSI}=\left(RSI_{curr}-RSI_{L_{Low_{10}}}\right)/\left(RSI_{H_{High_{10}}}-RSI_{L_{Low_{10}}}\right)$

where $RSI_{L_{Low_{10}}}$ and $RSI_{H_{High_{10}}}$ are the lowest low and highest high of the last ten RSI values.

*T3-triple exponential moving average*. A triple exponential moving average [54] is a moving average indicator where the main motivation for its development is to reduce lag in the time series response. For this reason, we use the closing prices for our calculation and perform a reversal explanation calculation as follows:

- *T*3 = *c*_1_ × *EMA*_6_ + *c*_2_ × *EMA*_5_ + *c*_3_ × *EMA*_4_ + *c*_4_ × *EMA*_3_

with:

- *c*_1_ = −*α*^3^
- *c*_2_ = 3 × *α*^2^+ 3 × *α*^3^
- *c*_3_ = −6 × *α*^2^ − 3 × *α* − 3 × *α*^3^
- *c*_4_ = 1+ 3 × *α*+ *α*^3^+ 3 × *α*^2^
- *EMA*_1_ = *EMA*_10_(*CL*)
- *EMA*_2_ = *EMA*_10_(*EMA*_1_)
- *EMA*_3_ = *EMA*_10_(*EMA*_2_)
- *EMA*_4_ = *EMA*_10_(*EMA*_3_)
- *EMA*_5_ = *EMA*_10_(*EMA*_4_)
- *EMA*_6_ = *EMA*_10_(*EMA*_5_)

where *α* is the volume factor, and *EMA*_10_(*CL*) is the exponential moving average of the 10 previous 10-MB closing prices.

*Triple exponential moving average*. A triple exponential moving average (TEMA) [48] is an attempt to reduce the lag associated with MA by adding weight to the most recent prices:

- *TEMA* = (3 × *EMA*_10_(*CL*)) − (3 × *EMA*_10_(*EMA*_10_(*CL*))+ *EMA*_10_(*EMA*_10_(*EMA*_10_(*CL*)))

with EMA being, in every case, the exponential moving average of the previous 10 prices (i.e. previous EMA and closing prices).

*Triangular moving average*. A triangular moving average (TRIMA) is the average of the time series with emphasis placed on the middle region:

- *TRIMA* = *SMA*_10_(*SMA*_10_(*SMA*_10_(*CL*)))

where, for its calculation, we use the closing prices of the last 10 10-MB blocks.

*Triple exponential average*. A triple exponential average (TRIX) is a momentum oscillator which measures the rate of change of the triple smoothed moving average as follows:

- *EMA*_*First*_ = *EMA*_10_(*CL*)
- *EMA*_*Double*_ = *EMA*_10_(*EMA*_*First*_)
- *EMA*_*Triple*_ = *EMA*_10_(*EMA*_*Double*_)
- *TRIX* = 1-*period* *Rate* *of* *Change*.

*True strength index*. A true strength index (TSI) [55] is an indicator which specifies the overbought and oversold levels with market return anticipation. We calculate TSI as follows:

- *PC* = *CL*_*k*_ − *CL*_*k*−1_, where *k* = 2, …, *T*
- *APC* = |*CL*_*k*_ − *CL*_*k*−1_|, where *k* = 2, …, *T*
- *EMA*_1_ = *EMA*_25_(*PC*)
- *EMA*_2_ = *EMA*_13_(*EMA*_1_)
- *EMA*_3_ = *EMA*_25_(*APC*)
- *EMA*_4_ = *EMA*_13_(*EMA*_3_)
- *TSI* = 100 × *EMA*_2_/*EMA*_4_

where PC represents the closing price differences for the whole time series lookback period.

*Ultimate oscillator*. An ultimate oscillator (UO) [56] is a momentum oscillator indicator with a multiple timeframe perspective. There are three main modules as presented in the following calculations:

- Average of seven 10-MB blocks
  - $BP=CL_{t}-\left(CL_{t-1}1_{CL_{t-1}<L_{t}}+L_{t}1_{CL_{t-1}>L_{t}}\right)$
  - $TR_{1}=CL_{t-1}1_{CL_{t-1}>H_{t}}+H_{curr}1_{CL_{t-1}<H_{t}}$
  - $TR_{2}=CL_{t-1}1_{CL_{t-1}<L_{t}}+L_{t}1_{CL_{t-1}>L_{t}}$
  - *TR* = *TR*_1_ + *TR*_2_
  - $Average_{7}=\sum_{l=1}^{7}BP_{l}/\sum_{l=1}^{7}TR_{l}$
- Average of fourteen 10-MB blocks
  - $BP=CL_{t}-\left(CL_{t-1}1_{CL_{t-1}<L_{t}}+L_{t}1_{CL_{t-1}>L_{t}}\right)$
  - $TR_{3}=CL_{t-1}1_{CL_{t-1}>H_{t}}+H_{t}1_{CL_{t-1}<H_{t}}$
  - $TR_{4}=CL_{t-1}1_{CL_{t-1}<L_{t}}+L_{t}1_{CL_{t-1}>L_{t}}$
  - *TR* = *TR*_3_ + *TR*_4_
  - $Average_{14}=\sum_{l=1}^{14}BP_{l}/\sum_{l=1}^{14}TR_{l}$
- Average of twenty-eight 10-MB blocks
  - $BP=CL_{t}-\left(CL_{t-1}1_{CL_{t-1}<L_{t}}+L_{t}1_{CL_{t-1}>L_{t}}\right)$
  - $TR_{5}=CL_{t-1}1_{CL_{t-1}>H_{t}}+H_{t}1_{CL_{t-1}<H_{t}}$
  - $TR_{6}=CL_{t-1}1_{CL_{t-1}<L_{t}}+L_{t}1_{CL_{t-1}>L_{t}}$
  - *TR* = *TR*_5_ + *TR*_6_
  - $Average_{28}=\sum_{l=1}^{28}BP_{l}/\sum_{l=1}^{28}TR_{l}$
- *UO* = 100 × [(4 × *Average*_7_)+ (2 × *Average*_14_)+ *Average*_28_]/(4+ 2+ 1)

where *BP* represents buying pressure.

*Weighted close*. Weighted close (WCL) is the average of the four universal types of prices which are included in each of our 10-MB blocks:

- *WCL* = (*H*_*t*_ + *L*_*t*_+ 2 × *CL*_*t*_)/4.

*Williams %R*. Williams %R [56] is a momentum technical indicator which informs the ML trader whether the market is trading close to the high or low trading range. It is calculated as follows:

- $\%R=-100\times \left(H_{High_{14}}-CL_{t}\right)/\left(H_{High_{14}}-L_{Low_{14}}\right)$

where -100 corrects the inversion.

*Zero-lag exponential moving average*. Zero-lag exponential moving average (ZLEMA) belongs to the EMA family of indicators where the main purpose is to reduce or remove the impulse lag by introducing an error term. It is calculated as follows:

- *error* = *CL* − *CL*_*lag*_
- *Input* = *CL*+ *error*
- *ZLEMA* = *EMA*_10_(*Input*)

where *lag* = (*N* − 1)/2 with N = 1 in our case.

*Fractals*. A fractal [39] is an indicator used to detect top and bottom trends by focusing on five consecutive blocks, which, in our case, are five 10-MB blocks used for two different scenarios:

- *Buy* *Fractals*
  A buy fractal is a sequence of five consecutive 10-MB blocks where the highest high is preceded by two lower highs and is followed by two lower highs.
- *Sell* *Fractals*
  The opposite framework is a sell fractal. 10-MB blocks can overlap in the quest of these two types of fractals.

Here, we calculate fractals separately for the open, close, lowest, and highest 10-MB block prices.

*Linear regression line*. Linear regression line (LRL) is a basic statistical method that provides information for a future projection wherein trading is used to capture overextended price trends. We perform LRL for each 10-MB block without any prior stationarity assumptions. The basic calculations are as follows:

- *PV* = *c*_1_ + *c*_2_ × *MB*_*prices*_
- $c_{2}=r\times (std_{PV}/std_{MB_{prices}})$
- $r=\frac{\left(\sum_{i=1}^{10}\left(MB_{prices}\left(i\right)-\bar{MB_{prices}}\right)\left(PV\left(i\right)-\bar{PV}\right)\right)}{(\sqrt{\sum_{i=1}^{10}{\left(MB_{prices}\left(i\right)-\bar{MB_{prices}}\right)}^{2}(\sum_{i=1}^{10}{\left(PV\left(i\right)-\bar{PV}\right)}^{2})}}$
- $c_{1}=\bar{PV}-c_{2}\times \bar{MB_{prices}}$

where *PV* are the predicted values, r is the correlation coefficient, and $\bar{MB_{prices}}$ and $\bar{PV}$ are the mean of 10-MB block prices and predicted values, respectively.

*Digital filtering: Rational transfer function*. A rational transfer function [57] is a representation of a linear time-invariant (LTI) filter, with the assumption that the input signal depends on the time-frequency domain, which describes the input-output relationship of a signal. In the Z-tranform domain, we have the following rational transfer function:

- $O(z)=\frac{b\left(1\right)+b\left(2\right)z^{-1}+...+b\left(n_{b}+1\right)+z^{-n_{b}}}{1+\alpha \left(2\right)z^{-1}+...+\alpha \left(n_{a}+1\right)z^{-n_{\alpha }}}I\left(z\right)$,

where:

- *I*(*z*) and *O*(*z*) are the input (i.e. 10-MB block closing prices) and output respectively,
- *b* are the numerator coefficients,
- *α* are the denominator coefficients,
- *n*_*a*_ is the feedback order,
- *n*_*b*_ is the feedforward order,
- *z* is the complex variable,
- the lookback period for the calculations is ten 10-MB blocks.

*Digital filtering: Savitzky-golay filter*. A Savitzky-Golay (S-G) digital filter [58, 59] is a discrete convolution with a specific impulse response. We describe how the ML trader can obtain the S-G signal based on higher degree polynomials:

- Least-Square Filter
  - Objective: minimize error $E_{N}=\sum_{i=l}^{m}w_{i}{\left(y_{i}-\sum_{r=0}^{n}p_{r}x_{i}^{r}\right)}^{2}$
  - Partial derivative of the polynomial coefficients:
    $$\frac{\partial Q}{\partial p_{k}}=0\to \sum_{i=l}^{m}w_{i}\sum_{r=0}^{n}p_{r}x_{i}^{r+k}=\sum_{i=l}^{m}w_{i}y_{i}x_{i}^{k}$$
  - Finite time series allow order summation change:
    $$\sum_{r=0}^{n}p_{r}\sum_{i=l}^{m}w_{i}x_{i}^{r+k}=\sum_{i=l}^{m}w_{i}y_{i}x_{i}^{k}$$
  - As a result, the desired linear equations are the following:
    $$\left[\begin{array}{cccc} \sum_{i=l}^{m}w_{i}x_{i}^{0} & \sum_{i=l}^{m}w_{i}x_{i}^{1} & \ldots & \sum_{i=l}^{m}w_{i}x_{i}^{n}\\ \sum_{i=l}^{m}w_{i}x_{i}^{1} & \sum_{i=l}^{m}w_{i}x_{i}^{2} & \ldots & \sum_{i=l}^{m}w_{i}x_{i}^{n+1}\\ \vdots & \vdots & \ddots & \vdots \\ \sum_{i=l}^{m}w_{i}x_{i}^{n} & \sum_{i=l}^{m}w_{i}x_{i}^{n+1} & \ldots & \sum_{i=l}^{m}w_{i}x_{i}^{2n} \end{array}\right]\left[\begin{array}{c} p_{0}\\ p_{1}\\ \vdots \\ p_{n} \end{array}\right]=\left[\begin{array}{c} \sum_{i=l}^{m}w_{i}y_{i}x_{i}^{0}\\ \sum_{i=l}^{m}w_{i}y_{i}x_{i}^{1}\\ \vdots \\ \sum_{i=l}^{m}w_{i}y_{i}x_{i}^{n} \end{array}\right]$$
    equivalent to the notation **A** **P** = **B** where matrix $A^{-1}\in R^{\left(n+1)\times (n+1\right)}$ under the condition that the polynomial degree is *n* ≤ *m* − *l*.
- S-G Filter
  - Local convolution coefficients calculation
    $$\begin{array}{c} \left[\begin{array}{c} p_{0}\\ p_{1}\\ \vdots \\ p_{n} \end{array}]={\left[\begin{array}{ccc} \sum_{i=l}^{m}w_{i}x_{i}^{0} & \ldots & \sum_{i=l}^{m}w_{i}x_{i}^{n}\\ \sum_{i=l}^{m}w_{i}x_{i}^{1} & \ldots & \sum_{i=l}^{m}w_{i}x_{i}^{n+1}\\ \vdots & \ddots & \vdots \\ \sum_{i=l}^{m}w_{i}x_{i}^{n} & \ldots & \sum_{i=l}^{m}w_{i}x_{i}^{2n} \end{array}\right]}^{-1}[\begin{array}{c} \sum_{i=l}^{m}w_{i}y_{i}x_{i}^{0}\\ \sum_{i=l}^{m}w_{i}y_{i}x_{i}^{1}\\ \vdots \\ \sum_{i=l}^{m}w_{i}y_{i}x_{i}^{n} \end{array}\right]\Rightarrow \\ \left[\begin{array}{c} p_{0}\\ p_{1}\\ \vdots \\ p_{n} \end{array}]=[\begin{array}{cccc} c_{0,0} & c_{0,1} & \ldots & c_{0,n}\\ c_{1,0} & c_{1,1} & \ldots & c_{1,n}\\ \vdots & \vdots & \ddots & \vdots \\ c_{n,0} & c_{n,1} & \ldots & c_{n,n} \end{array}][\begin{array}{c} \sum_{i=l}^{m}w_{i}y_{i}x_{i}^{0}\\ \sum_{i=l}^{m}w_{i}y_{i}x_{i}^{1}\\ \vdots \\ \sum_{i=l}^{m}w_{i}y_{i}x_{i}^{n} \end{array}\right] \end{array}$$
  - Response at the local point of 0 degree is:
    $$y[0]=c_{0,0}*\sum_{i=l}^{m}w_{i}y_{i}x_{i}^{0}+c_{0,1}*\sum_{i=l}^{m}w_{i}y_{i}x_{i}^{1}+...+c_{0,n}*\sum_{i=l}^{m}w_{i}y_{i}x_{i}^{n}$$

*Digital filtering: Zero-phase filter*. A zero-phase filter [60] is a bidirectional filtering technique. With zero phase slope and even impulse response *h*(*n*), the filter provides an output signal, which is a zero phase recursive signal. This method is suitable for our experimental protocol since we use training and testing sets rather than online learning architecture as we will do in 4.2.4. The calculation process is as follows:

- Real Impulse response: *h*(*n*), n∈Z
- Discrete-time Fourier Transformation:
  $$H_{\omega T}\left(h\right)=\sum_{n=1}^{\infty }h\left(n\right)cos\left(\omega nT\right)-j\sum_{n=1}^{\infty }h\left(n\right)sin\left(\omega nT\right)$$
- Based on Euler formula and *h*-even: $H\left(e^{j\omega T}\right)=\sum_{n=1}^{\infty }h\left(n\right)cos\left(\omega nT\right)$

*Remove offset and detrend*. We present three detrend methods for short-term cycle isolation and calculate them as follows:

- Remove Offset
  - $Offset=CL_{t}-\left(\sum_{l=1}^{n}CL_{l}\right)/n$
    where *n* denotes the 10-MB lookback period
- Detrend—Least Squares Fitting Line
  - $R^{2}=\sum_{i=1}^{n}{\left[y_{i}-g\left(x_{i}\right)\right]}^{2}$
  - $\frac{\partial (R^{2})}{\partial \alpha }=0$
  - $\frac{\partial (R^{2})}{\partial b}=0$
  - $\left[\begin{array}{c} \alpha \\ b \end{array}]={\left[\begin{array}{cc} n & \sum_{i=l}^{n}x_{i}\\ \sum_{i=l}^{n}x_{i} & \sum_{i=l}^{n}x_{i}^{2} \end{array}\right]}^{-1}[\begin{array}{c} \sum_{i=l}^{n}y_{i}\\ \sum_{i=l}^{n}x_{i}y_{i} \end{array}\right]$
    where *α* and *b* are the regression coefficients of *g*, and *x* represents the 10-MB closing prices.

*Beta-like calculation*. Beta [61] is a volatility indicator which considers market risk. We adjust the notion of beta calculation to our experimental protocol where we index based on the average of the closing prices (*Av*_*CL*_) with *Av*_*t*_ as the current MB block price and *Av*_*t*−1_ as the previous MB block’s closing price. Our calculations are as follow:

- *Index*_*CL*_ = *CL*_*t*_/*CL*_*t*−1_
- $Index_{Av_{CL}}=Av_{t}/Av_{t-1}$
- *Dev*_*CL*_ = *Index*_*CL*_ − *SMA*_10_(*Index*_*CL*_)
- $Dev_{Av_{CL}}=Index_{Av_{CL}}-SMA_{10}\left(Index_{Av_{CL}}\right)$
- $Beta=cov_{10}\left(Dev_{CL},Dev_{Av_{CL}}\right)/var_{10}\left(Dev_{Av_{CL}}\right)$

where $cov_{10}\left(Dev_{CL},Dev_{Av_{CL}}\right)$ represents the covariance between the current closing price and the average of the previous ten 10-MB closing prices, and $var_{10}\left(Dev_{Av_{CL}}\right)$ is the variance of the sum of the ten previous $Index_{Av_{CL}}$.

#### Quantitative analysis

Quantitative analysis captures trading activity mainly via statistical modelling. We focus on time series analysis, and more specifically, we examine features such as autocorrelation and partial autocorrelation, among others (e.g., statistical tests), while at the end of the section, we build an ML feature extraction method based on an online learning setup and test the validity of our hypothesis.

*Autocorrelation and partial correlation*. Autocorrelation and partial correlation [62, 63] are key features in the development of time series analysis. We treat our time series (i.e. stock prices and log returns per 10-MB blocks) as stationary stochastic processes since we estimate their local behavior based on 10-MB blocks:

- Autocorrelation
  - $ac_{k}=\frac{E[\left(z_{t}-\mu \right)\left(z_{t+k}-\mu \right)]}{\sqrt{E\left[{\left(z_{t}-\mu \right)}^{2}\right]E\left[{\left(z_{t+k}-\mu \right)}^{2}\right]}}$
  where *z*_*t*_ and *z*_*t*+ *k*_ are the time series of lag *k*, $\mu =E\left[z_{t}\right]=\int_{-\infty }^{\infty }zp\left(z\right)dz$ and $\sigma_{z}^{2}=E\left[{\left(z_{t}-\mu \right)}^{2}\right]=\int_{-\infty }^{\infty }{\left(z-\mu \right)}^{2}p\left(z\right)dz$ are the constant mean and constant variance respectively.
- Partial Correlation
  - For the general case of an autoregressive model *AR*(*p*), we have:
    *x*_*i*+ 1_ = *ϕ*_1_ *x*_*i*_ + *ϕ*_2_ *x*_*i*−1_+ …+ *ϕ*_*p*_ *x*_*i*−*p*+ 1_ + *ξ*_*i*+ 1_ of lag 1 up to *p* follows:
    - $<x_{i}x_{i+1}>=\sum_{j=1}^{p}\left(\phi_{j}<x_{i}x_{i-j+1}>\right)$
    - $<x_{i-1}x_{i+1}>=\sum_{j=1}^{p}\left(\phi_{j}<x_{i-1}x_{i-j+1}>\right)$
    - $<x_{i-k+1}x_{i+1}>=\sum_{j=1}^{p}\left(\phi_{j}<x_{i-k+1}x_{i-j+1}>\right)$
    - $<x_{i-p+1}x_{i+1}>=\sum_{j=1}^{p}\left(\phi_{j}<x_{i-p+1}x_{i-j+1}>\right)$
      by diving with *N* − 1 and autocovariance of zero separated periods (where the autocovariance function is even), all the lag periods above will be:
    - $r_{1}=\sum_{j=1}^{p}\phi_{j}r_{j-1}$
    - $r_{2}=\sum_{j=1}^{p}\phi_{j}r_{j-2}$
    - $r_{k}=\sum_{j=1}^{p}\phi_{j}r_{j-k}$
    - $r_{p}=\sum_{j=1}^{p}\phi_{j}r_{j-p}$
    where *r*_1_, *r*_2_, …, *r*_*k*_, *r*_*p*_ can be described by the matrix operations ***R*Φ** = ***r***, $R\in R^{p\times p}$, $\Phi \in R^{p\times 1}$ and $r\in R^{p\times 1}$. The symmetric and full rank ***Φ*** are as follows: $\hat{\Phi }=R^{-1}r$.
  - *Yule-Walker* Equations calculation:
    - Lag interval 1 ≤ *i* ≤ *p*
    - $\hat{\Phi }={\left(R^{\left(i\right)}\right)}^{-1}r^{\left(i\right)}=[\begin{array}{c} \hat{\phi_{1}}\\ \hat{\phi_{2}}\\ \vdots \\ \hat{\phi_{i}} \end{array}]$

*Cointegration*. We investigate time-series equilibrium [64, 65] by testing the cointegrated hypothesis. Utilizing the cointegration test will help ML traders avoid the problem of spurious regression. We employ the Engle-Granger (EG) test for the multivariable case of LOB ask (*A*_*t*_) and bid (*B*_*t*_) times series. We formulate the EG test for the ask and bid LOB prices as follows:

- *A*_*t*_ and *B*_*t*_
∼
*I*(*d*), where *I*(*d*) represents the order of integration
- Cointegration equation based on the error term: *u*_*t*_ = *A*_*t*_ − *αB*_*t*_
- EG Hypothesis: *u*(*t*)∼
*I*(*d*), *d* ≠ 0
- Perform ordinary leat squares (OLS) for the estimation of $\hat{\alpha }$ and unit root test for: $\hat{u}=A_{t}-\hat{\alpha }B_{t}$

*Order book imbalance*. We calculate the order book imbalance [4] based on the volume depth of our LOB as follows:

- $VI=\frac{V_{l}^{b}-V_{l}^{\alpha }}{V_{l}^{b}+V_{l}^{\alpha }}$

where $V_{l}^{\alpha }$ and $V_{l}^{b}$ are the volume sizes for the ask and bid LOB sides at level *l*.

### Additional results

Tables 5–9 are provided below:

**Table 5:** Results based on the hand-crafted features based on LOB features—136 features. Bold text highlights the best F1 performance per predicted horizon T. LMS classifier achieved the best F1 performance for every predicted horizon.

Sorting	Classifier	T	*Accuracy*	*Precision*	*Recall*	*F*1
Entropy	LMS	10	0.420 ± 0.025	0.379 ± 0.011	0.397 ± 0.011	0.355 ± 0.013
LMS1	LMS	10	0.574 ± 0.055	0.402 ± 0.013	0.396 ± 0.018	0.384 ± 0.020
LMS2	LMS	10	0.519 ± 0.015	0.400 ± 0.009	0.413 ± 0.009	**0.396** ± 0.010
LDA1	LDA	10	0.561 ± 0.090	0.389 ± 0.018	0.382 ± 0.016	0.363 ± 0.030
LDA2	LDA	10	0.507 ± 0.041	0.373 ± 0.014	0.384 ± 0.017	0.362 ± 0.019
Entropy	LMS	20	0.386 ± 0.018	0.386 ± 0.015	0.397 ± 0.015	0.363 ± 0.016
LMS1	LMS	20	0.527 ± 0.027	0.411 ± 0.013	0.389 ± 0.015	0.375 ± 0.029
LMS2	LMS	20	0.462 ± 0.013	0.405 ± 0.013	0.410 ± 0.009	**0.400** ± 0.012
LDA1	LDA	20	0.529 ± 0.031	0.406 ± 0.017	0.381 ± 0.011	0.360 ± 0.024
LDA2	LDA	20	0.461 ± 0.036	0.378 ± 0.016	0.380 ± 0.016	0.368 ± 0.021
Entropy	LMS	30	0.391 ± 0.016	0.395 ± 0.018	0.401 ± 0.015	0.380 ± 0.017
LMS1	LMS	30	0.459 ± 0.025	0.405 ± 0.017	0.388 ± 0.020	0.366 ± 0.040
LMS2	LMS	30	0.432 ± 0.009	0.407 ± 0.015	0.409 ± 0.013	**0.401** ± 0.016
LDA1	LDA	30	0.447 ± 0.041	0.391 ± 0.018	0.377 ± 0.017	0.352 ± 0.037
LDA2	LDA	30	0.418 ± 0.028	0.373 ± 0.016	0.375 ± 0.015	0.361 ± 0.018

**Table 6:** Results based only on technical indicators—82 features. Bold text highlights the best F1 performance per predicted horizon T. LMS classifier achieved the best F1 performance for every predicted horizon.

Sorting	Classifier	T	*Accuracy*	*Precision*	*Recall*	*F*1
Entropy	LMS	10	0.456 ± 0.038	0.372 ± 0.021	0.380 ± 0.014	0.353 ± 0.020
LMS1	LMS	10	0.497 ± 0.066	0.371 ± 0.017	0.377 ± 0.021	0.354 ± 0.024
LMS2	LMS	10	0.460 ± 0.016	0.383 ± 0.012	0.394 ± 0.009	**0.365** ± 0.010
LDA1	LDA	10	0.517 ± 0.064	0.367 ± 0.015	0.371 ± 0.020	0.344 ± 0.026
LDA2	LDA	10	0.475 ± 0.023	0.371 ± 0.010	0.382 ± 0.009	0.351 ± 0.015
Entropy	LMS	20	0.430 ± 0.029	0.384 ± 0.025	0.387 ± 0.017	0.371 ± 0.023
LMS1	LMS	20	0.480 ± 0.033	0.384 ± 0.023	0.381 ± 0.021	0.364 ± 0.037
LMS2	LMS	20	0.452 ± 0.011	0.400 ± 0.018	0.402 ± 0.011	**0.391** ± 0.015
LDA1	LDA	20	0.483 ± 0.034	0.379 ± 0.022	0.377 ± 0.020	0.355 ± 0.038
LDA2	LDA	20	0.453 ± 0.014	0.382 ± 0.015	0.387 ± 0.009	0.369 ± 0.016
Entropy	LMS	30	0.423 ± 0.030	0.394 ± 0.028	0.394 ± 0.020	0.385 ± 0.027
LMS1	LMS	30	0.450 ± 0.018	0.395 ± 0.028	0.393 ± 0.027	0.379 ± 0.050
LMS2	LMS	30	0.446 ± 0.013	0.409 ± 0.020	0.408 ± 0.013	**0.403** ± 0.019
LDA1	LDA	30	0.430 ± 0.041	0.384 ± 0.027	0.382 ± 0.027	0.353 ± 0.053
LDA2	LDA	30	0.433 ± 0.021	0.397 ± 0.017	0.396 ± 0.016	0.386 ± 0.026

**Table 7:** Results based on quantitative features—55 features. Bold text highlights the best F1 performance per predicted horizon T. LMS classifier achieved the best F1 performance for every predicted horizon.

Sorting	Classifier	T	*Accuracy*	*Precision*	*Recall*	*F*1
Entropy	LMS	10	0.393 ± 0.109	0.399 ± 0.047	0.419 ± 0.047	0.340 ± 0.064
LMS1	LMS	10	0.665 ± 0.033	0.468 ± 0.043	0.388 ± 0.016	0.366 ± 0.016
LMS2	LMS	10	0.571 ± 0.071	0.470 ± 0.053	0.418 ± 0.032	**0.384** ± 0.020
LDA1	LDA	10	0.611 ± 0.088	0.422 ± 0.039	0.390 ± 0.020	0.370 ± 0.024
LDA2	LDA	10	0.380 ± 0.101	0.401 ± 0.024	0.428 ± 0.027	0.339 ± 0.063
Entropy	LMS	20	0.400 ± 0.074	0.408 ± 0.048	0.422 ± 0.048	0.372 ± 0.061
LMS1	LMS	20	0.553 ± 0.029	0.429 ± 0.037	0.373 ± 0.016	0.335 ± 0.025
LMS2	LMS	20	0.483 ± 0.017	0.447 ± 0.022	0.457 ± 0.025	**0.435** ± 0.029
LDA1	LDA	20	0.513 ± 0.072	0.402 ± 0.032	0.379 ± 0.026	0.347 ± 0.040
LDA2	LDA	20	0.424 ± 0.073	0.431 ± 0.026	0.444 ± 0.023	0.340 ± 0.053
Entropy	LMS	30	0.410 ± 0.062	0.416 ± 0.051	0.423 ± 0.048	0.391 ± 0.060
LMS1	LMS	30	0.478 ± 0.022	0.407 ± 0.038	0.370 ± 0.019	0.320 ± 0.036
LMS2	LMS	30	0.481 ± 0.012	0.457 ± 0.026	0.460 ± 0.024	**0.449** ± 0.034
LDA1	LDA	30	0.464 ± 0.037	0.394 ± 0.030	0.378 ± 0.027	0.338 ± 0.049
LDA2	LDA	30	0.425 ± 0.063	0.437 ± 0.029	0.443 ± 0.022	0.406 ± 0.055

**Table 8:** F1 based on different numbers of best features for the five criteria. Bold text highlights the best F1 performance for the predicted horizon T = 10. LMS classifier achieved the best F1 performance for every predicted horizon.

Sorting	Classifier	5	50	100	200	273
Entropy	LMS	0.319 ± 0.008	0.363 ± 0.027	0.414 ± 0.020	0.425 ± 0.025	0.440 ± 0.018
LMS1	LMS	0.377 ± 0.008	0.374 ± 0.036	0.393 ± 0.047	0.419 ± 0.036	0.440 ± 0.018
LMS2	LMS	0.402 ± 0.015	0.443 ± 0.014	**0.441** ± 0.018	**0.440** ± 0.018	0.440 ± 0.018
LDA1	LMS	0.373 ± 0.013	0.380 ± 0.017	0.395 ± 0.016	0.315 ± 0.018	0.289 ± 0.025
LDA2	LMS	0.412 ± 0.011	0.420 ± 0.017	0.420 ± 0.019	0.289 ± 0.013	0.309 ± 0.027
LDA1	LMS	0.370 ± 0.011	0.372 ± 0.032	0.387 ± 0.041	**0.440** ± 0.017	0.440 ± 0.018
LDA2	LMS	**0.421** ± 0.010	0.435 ± 0.011	0.435 ± 0.014	**0.440** ± 0.017	0.441 ± 0.018
Entropy	RBFN	0.316 ± 0.010	0.363 ± 0.020	0.413 ± 0.016	0.430 ± 0.016	0.441 ± 0.016
LMS1	RBFN	0.387 ± 0.018	0.402 ± 0.022	0.421 ± 0.017	0.429 ± 0.017	0.441 ± 0.016
LMS2	RBFN	0.403 ± 0.015	**0.444** ± 0.012	0.439 ± 0.013	0.439 ± 0.019	0.442 ± 0.016
LDA1	RBFN	0.371 ± 0.011	0.387 ± 0.021	0.411 ± 0.013	**0.440** ± 0.016	0.441 ± 0.016
LDA2	RBFN	0.416 ± 0.011	0.434 ± 0.015	0.436 ± 0.015	0.436 ± 0.016	**0.442** ± 0.016

**Table 9:** List for the first 10 best features for the 5 sorting methods.

Feature Sets	Description
Entropy	
1	Autocorrelation
2	Donchian Channels
3	Highest High
4	Center of Gravity Oscillator
5	Heikin-Ashi
6	Linear Regression—Regression Coeffic.
7	Linear Regression—Correlation Coeffic.
8	T3
9	TEMA
10	TRIMA
LMS1	
1	Logistic Regression—Local Spatial Ratio
2	Best LOB Level—Bid Side Volume
3	Second Best LOB Level—Ask Volume
4	Price and Volume Derivation
5	Best LOB Level—Ask Side
6	Linear Regression—Correlation Coeffic.
7	Logistic Regression—Logistic Coeffic.
8	Logistic Regression—Extended Spatial Ratio
9	Autocorrelation for Log Returns
10	Partial Autocorrelation
LMS2	
1	Logistic Regression—Spatial Ratio
2	Cointegration—Boolean Vector
3	Cointegration—Test Statistics
4	Price and Volume Means
5	Average Type Intensity
6	Average Type Intensity
7	Spread & Mid-Price
8	Alligator Jaw
9	Directional Index
10	Fractals
LDA1	
1	Logistic Regression—Spatial Ratio
2	Second Best LOB Level—Ask Volume
3	Price & Volume derivation
4	Spread & Mid-Price
5	Partial Autocorrelation for Log Returns
6	Linear Regression Line—Squared Correlation Coeffic.
7	Order Book Imbalance
8	Linear Regression—Correlation Coeffic.
9	Linear Regression—Regression Coeffic.
10	Third Best LOB Level—Ask Volume
LDA2	
1	Logistic Regression—Probability Estimation
2	Logistic Regression—Spatial Ratio
3	Bollinger Bands
4	Alligator Teeth
5	Cointegration—Test Statistics
6	Best LOB Level—Bid Side Volume
7	Cointegration—p Values
8	Price & Volume Means
9	Price & Volume Derivation
10	Price Differences

### Feature sorting lists

These are the five sorted lists of the 273 hand-crafted features. A detailed feature name list follows.

1. Features sorting list based on Entropy:
  {218;161;170;160;166;206;209;196;199;197;193;174;182;183;146;157;171;172;173;156;155;184;194;137;177;176;138;195;136;217;213;181;147;245;243;247;188;241;242;244;240;246;255;248;249;189;210;265;211;226;236;225;235;221;231;223;233;222;232;214;169;220;230;228;238;224;234;83;84;227;237;139;135;134;142;162;140;185;129;86;128;186;212;141;250;261;16;20;262;207;153;14;81;12;208;260;4;18;10;259;24;196;2;163;150;187;82;28;192;22;8;26;6;32;30;36;34;40;148;175;154;149;38;151;60;59;58;57;56;55;54;53;52;51;198;215;216;158;167;159;168;152;164;100;102;21;25;29;13;17;9;5;33;1;23;19;15;27;11;31;37;7;3;35;85;39;104;252;96;98;106;269;108;92;112;110;88;253;116;124;120;145;94;50;90;180;256;114;49;165;118;123;122;48;126;268;119;115;80;47;111;143;144;46;107;70;45;125;103;121;44;117;43;113;99;42;109;254;270;271;105;41;95;101;91;272;97;273;93;179;69;178;87;68;79;89;127;67;78;201;191;66;133;77;65;76;205;203;61;200;202;71;75;62;72;64;206;204;257;63;132;131;74;73;130;251;258;267;266;219;229;239;263;264}
2. Features sorting list based on LMS1:
  {266;4;6;87;1;209;267;268;218;254;251;92;253;250;91;252;255;193;122;270;174;183;267;108;103;32;18;147;216;100;118;263;264;111;41;90;112;20;273;24;127;116;120;109;110;126;225;235;164;107;98;102;124;165;89;94;133;114;119;104;96;95;88;115;188;61;93;125;101;97;105;113;208;211;99;180;121;117;246;106;123;189;248;228;238;8;210;186;130;185;10;14;242;157;136;16;249;240;244;65;66;64;69;153;28;73;22;170;143;142;184;178;30;154;76;79;67;75;63;74;78;256;247;245;146;219;229;239;68;187;62;70;176;201;179;194;72;197;131;132;77;217;42;43;44;45;46;47;48;49;50;71;85;207;40;258;226;236;80;260;262;134;135;36;204;144;145;38;26;12;84;199;195;182;215;156;171;158;151;167;148;161;168;191;152;159;160;149;150;141;198;169;166;2;212;213;181;3;5;7;9;11;13;15;17;19;21;23;25;27;29;31;33;35;37;39;51;52;53;54;55;56;57;58;59;60;81;82;86;155;163;196;175;214;272;172;173;140;190;192;139;200;162;227;237;222;232;220;230;241;243;224;234;271;206;34;83;177;205;203;221;223;231;233;202;138;137;261;128;129;257;259;265}
3. Features sorting list based on LMS2:
  {269;257;262;81;127;128;41;142;181;205;203;259;223;202;200;199;243;273;176;206;256;204;265;132;10;2;14;84;170;78;240;226;182;157;61;80;242;217;49;70;50;207;165;150;164;93;87;62;43;89;66;215;18;154;251;111;222;8;261;201;258;270;271;65;96;151;216;272;210;186;124;120;153;94;187;92;211;117;109;101;162;166;29;213;184;185;198;195;129;146;191;192;193;196;174;171;159;149;161;139;125;113;106;102;266;118;104;218;36;38;156;190;250;63;85;133;12;121;90;34;40;175;91;248;241;227;245;152;130;189;178;214;136;137;158;224;225;112;99;115;264;212;169;141;163;220;221;188;197;194;209;208;168;22;105;114;110;268;16;23;177;140;119;123;100;126;122;260;244;246;134;135;131;56;103;173;167;6;228;47;97;255;107;180;71;155;4;254;253;179;82;138;32;28;143;252;116;30;144;147;88;108;73;95;98;249;20;51;160;247;55;59;5;148;42;7;76;31;54;3;145;77;46;19;231;48;17;15;83;232;21;45;52;230;236;37;1;24;58;69;13;53;35;67;172;33;183;79;86;26;267;75;219;25;234;9;44;39;11;229;237;57;235;239;60;27;68;64;74;233;238;72;263}
4. Features sorting list based on LDA1:
  {269;6;88;43;240;210;265;209;206;9;252;251;253;268;8;108;114;174;193;254;100;263;264;110;186;273;216;90;99;122;185;92;183;267;16;225;235;14;103;119;112;107;95;104;147;111;91;115;270;127;109;116;120;18;89;94;118;126;98;180;106;208;250;124;96;188;113;125;121;153;123;105;117;93;97;101;248;242;61;133;189;87;102;211;145;66;65;64;69;136;184;142;73;76;157;75;74;78;67;63;79;170;178;77;219;229;239;262;182;130;245;70;22;194;244;24;12;266;84;247;167;173;146;60;207;59;17;33;196;158;165;4;218;25;149;203;3;36;53;37;86;21;30;155;58;164;48;246;161;223;26;85;226;205;41;144;80;47;15;135;179;152;27;160;39;38;81;241;50;236;40;220;7;204;260;83;143;258;168;166;51;141;162;23;57;19;131;10;42;132;82;56;49;62;154;128;5;228;259;55;181;191;163;156;187;272;213;224;52;46;35;1;54;234;169;150;255;227;45;238;31;201;192;190;199;261;172;44;134;2;140;129;20;72;214;215;195;68;151;271;198;237;171;11;29;137;221;222;32;13;217;148;230;232;231;233;197;28;159;249;139;212;256;176;177;243;71;200;34;138;175;202;257}
5. Features sorting list based on LDA2:
  {272;269;150; 143;262;2;259;83;87;71;129;205;204;120;6;48;248;141;179;203;212;139;184;43;144;118;79;177;18;52;8;193;132;110;70;191;100;103;146;241;138;206;252;247;273;63;211;207;22;10;142;244;16;258;122;221;219;108;217;47;93;140;40;180;202;34;256;4;115;96;218;134;102;99;270;111;253;66;189;88;90;94;36;199;12;75;254;243;72;137;45;265;64;251;77;222;155;255;210;104;209;174;267;105;194;50;14;126;109;32;170;200;125;98;127;89;227;44;201;119;28;245;61;65;268;192;216;112;20;186;42;250;187;107;121;116;84;185;128;30;237;156;124;160;195;133;147;41;223;215;123;113;135;173;148;271;214;169;131;232;39;149;35;178;68;190;31;198;106;157;188;38;260;168;153;228;55;5;69;246;114;67;15;266;76;152;33;183;37;27;238;46;242;17;166;101;54;23;117;58;56;11;167;261;9;91;162;29;7;97;163;151;233;57;78;24;95;86;225;164;220;154;181;249;171;230;229;130;172;60;26;182;51;1;3;136;159;25;59;208;145;85;53;80;224;92;240;13;81;231;175;264;197;257;74;158;234;213;196;176;235;19;21;263;165;82;226;236;73;161;239;62;49}

Detailed feature list:

{**1-40**:Raw LOB Levels, **41-60**:Spread & Mid-Price, **61-80**:Price Differences, **81-84**:Price & Volume Means, **85-86**:Accumulated Differences, **87-126**:Price & Volume Derivations, **127-132**:Average Intensity Per Type, **132-136**:Relative Intensity Comparison & Limit Activity Acceleration LOB features: Some of the trade types in Relative Intensity Comparison and Limit Activity Acceleration features do not appear in the Nordic dataset., **137**:Accumulation Distribution Line, **138**:Awesome Oscillator, **139**:Accelerator Oscillator, **140**:Average Directional Index, **141**:Average Directional Movement Index Rating, **142**:AlligatorsJaw, **143**:AlligatorsTeeth, **144**:AlligatorsLips, **145**:Absolute Price Oscillator, **146**:Aroon Indicator/AroonUp, **147**:Aroon Indicator/AroonDown, **148**:Aroon Oscillator, **149**:Average True Range, **150**:Bollinger Bands,**151**:Ichimoku Clouds/Tenkan sen, **152**:Ichimoku Clouds/Kijun sen, **153**:Ichimoku Clouds/Senkou Span, **154**:Ichimoku Clouds/Senkou Span 52-Period, **155**:Ichimoku Clouds/Chickou Span, **156**:Chande Momentum Oscillator, **157**:Chaikin Oscillator, **158**:Chandelier Exit Long, **159**:Chandelier Exit Short, **160**:Center of Gravity Oscillator, **161**:Donchian Channels/Upper Channel, **162**:Donchian Channels/Lower Channel, **163**:Donchian Channels/Middle Channel, **164**:Double Exponential Moving Average, **165**:Detrended Price Oscillator, **166**:Heikin Ashi Close, **167**:Heikin Ashi Open, **168**:Heikin Ashi High, **169**:Heikin Ashi Low, **170**:Highest High, **171**:Lowest Low, **172**:Hull Moving Average, **173**:Internal Bar Strength, **174**:Keltern Channels/Lower Channel, **175**:Keltern Channels/Middle Line, **176**:Keltern Channels/Upper Channel, **177**:Moving Average Convergence Divergence, **178**:Median Price based on High and Low, **179**:Positive Directional Index, **180**:Negative Directional Index, **181**:Positive Directional Index, **182**:Positive Directional Movement, **183**:Negative Directional Movement, **184**:Momentum, **185**:Variable Moving Average, **186**:Normalized Average True Range, **187**:Percentage Price Oscillator/Moving Average Convergence Divergence, **188**:Percentage Price Oscillator, **189**:Rate of Change, **190**:Relative Strength Index, **191**:Relative Strength Index without the 100^th^ extreme case, **192**:Parabolic Stop And Reverse/Rising Stop And Reverse, **193**:Parabolic Stop And Reverse/Folliwng Stop And Reverse:, **194**:Standard Deviation, **195**:Stochastic Relative Strength Index, **196**:T3 Triple Exponential Moving Average, **197**:Triple Exponential Moving Average, **198**:Triangular Moving Average, **199**:Triple Exponential Average, **200**:True Strength Index, **201**:Ultimate Oscillator, **202**:Weighted Close Price, **203**:Williams %R, **204**:Weighted Moving Average, **205**:Fractals, **206**:Linear Regression Line/Regression Coefficient, **207**:Linear Regression Line/Intercept, **208**:Linear Regression Line/Slope, **209**:Linear Regression Line/Correlation Coefficient, **210**:Linear Regression Line/R-squared, **211**:Digital Filtering/Rational Transfer Function, **212**:Digital Filtering/Low-Pass Savitzky-Golay Filter, **213**:Zero-Phase digital filtering, **214**:Remove Offset, **215**:Remove Baseline, **216**:Detrend, **217**:Beta like Calculation, **218—254**:Autocorrelation & Partial Autocorrelation for LOB Levels, **255**:Correlation Between Price and Volume, **256**:Bollinger Bands, **257—258**:Cointegration Test/h Values, **259-260**:Cointegration Test/p values, **261-262**:Cointegration Test/Statistics, **263-264**:Cointegration Test/C Values, **265**:Order Book Imbalance, **266—273**:Logistic Regression.}

## References

[1] Dash R, Dash PK. A hybrid stock trading framework integrating technical analysis with machine learning techniques. The Journal of Finance and Data Science. 2016;2(1):42–57. 10.1016/j.jfds.2016.03.002

[2] Gould MD, Porter MA, Williams S, McDonald M, Fenn DJ, Howison SD. Limit order books. Quantitative Finance. 2013;13(11):1709–1742. 10.1080/14697688.2013.803148

[3] Passalis N, Tsantekidis A, Tefas A, Kanniainen J, Gabbouj M, Iosifidis A. Time-series classification using neural bag-of-features. IEEE 25th European Conference of Signal Processing; 2017. p. 301–305, 2017.

[4] Sirignano JA. Deep learning for limit order books. Quantitative Finance. 2019;19(4):549–570. 10.1080/14697688.2018.1546053

[5] Thanh DT, Kanniainen J, Gabbouj M, Iosifidis A. Tensor representation in high-frequency financial data for price change prediction. arXiv:170901268. 2017;.

[6] Tsantekidis A, Passalis N, Tefas A, Kanniainen J, Gabbouj M, Iosifidis A. Forecasting stock prices from the limit order book using convolutional neural networks. IEEE 19th Conference on Business Informatics. vol. 1; 2017. p. 7-12, 2017.

[7] Tsantekidis A, Passalis N, Tefas A, Kanniainen J, Gabbouj M, Iosifidis A. Using deep learning to detect price change indications in financial markets. IEEE 25th European Conference of Signal Processing; 2017. p. 2511–2515, 2017.

[8] Ntakaris A, Mirone G, Kanniainen J, Gabbouj M, Iosifidis A. Feature Engineering for Mid-Price Prediction With Deep Learning. IEEE Access. 2019;7:82390–82412. 10.1109/ACCESS.2019.2924353

[9] Chandrashekar G, Sahin F. A Survey on Feature Selection Methods. Comput Electr Eng. 2014;40(1):16–28. 10.1016/j.compeleceng.2013.11.024

[10] Miao J, Niu L. A survey on feature selection. Procedia Computer Science. 2016;91:919–926. 10.1016/j.procs.2016.07.111

[11] Battiti R. Using mutual information for selecting features in supervised neural net learning. IEEE Transactions on neural networks. 1994;5(4):537–550. 10.1109/72.298224 18267827

[12] Liu H, Sun J, Liu L, Zhang H. Feature selection with dynamic mutual. information. Pattern Recognition. 2009;42(7):1330–1339. 10.1016/j.patcog.2008.10.028

[13] Murphy JJ. Technical Analysis of the Financial Markets: A Comprehensive Guide to Trading Methods and Applications. New York Institute of Finance Series. New York Institute of Finance; 1999. Available from: https://books.google.fi/books?id=5zhXEqdr_IcC.

[14] Scholtus ML, van Dijk DJ. High-frequency technical trading: The importance of speed. Available at SSRN: https://ssrncom/abstract=2013789. 2012;.

[15] Kablan A, Ng W. High frequency trading using fuzzy momentum analysis. Proceedings of the World Congress on Engineering. vol. 1; 2010.

[16] Patel J, Shah S, Thakkar P, Kotecha K. Predicting stock and stock price index movement using Trend Deterministic Data Preparation and machine learning techniques. Expert Systems with Applications. 2015;42(1):259–268. 10.1016/j.eswa.2014.07.040

[17] Chan LKC, Karceski J, Lakonishok J. On Portfolio Optimization: Forecasting Covariances and Choosing the Risk Model. The Review of Financial Studies. 1999;12(5):937–974. 10.1093/rfs/12.5.937

[18] Perold AF. Large-Scale Portfolio Optimization. Management Science. 1984;30(10):1143–1160. 10.1287/mnsc.30.10.1143

[19] Fama EF. Risk, return and equilibrium: some clarifying comments. The Journal of Finance. 1968;23(1):29–40. 10.1111/j.1540-6261.1968.tb02996.x

[20] Sharpe WF. Capital asset prices: A theory of market equilibrium under conditions of risk. The Journal of Finance. 1964;19(3):425–442. 10.2307/2977928

[21] Diebold FX, Hahn J, Tay AS. Multivariate Density Forecast Evaluation and Calibration In Financial Risk Management: High-Frequency Returns on Foreign Exchange. The Review of Economics and Statistics. 1999;81(4):661–673. 10.1162/003465399558526

[22] Smith CW, Smithson CW, Wilford DS. Managing Financial Risk. Journal of Applied Corporate Finance. 1989;1(4):27–48. 10.1111/j.1745-6622.1989.tb00172.x

[23] Iosifidis A, Tefas A, Pitas I. Multidimensional sequence classification based on fuzzy distances and discriminant analysis. IEEE Transactions on Knowledge and Data Engineering. 2012;25(11):2564–2575. 10.1109/TKDE.2012.223

[24] Taylor SJ. Modelling financial time series. World Scientific; 2008.

[25] Shen S, Jiang H, Zhang T. Stock market forecasting using machine learning algorithms. Department of Electrical Engineering, Stanford University, Stanford, CA, Available at: https://wwwsemanticscholarorg/paper/Stock-Market-Forecasting-Using-Machine-Learning-Shen-Jiang/b68e8d2f4d2c709bb5919b82effcb6a7bbd3db37. 2012; p. 1–5.

[26] Poterba JM, Summers LH. Mean reversion in stock prices: Evidence and implications. Journal of financial economics. 1988;22(1):27–59. 10.1016/0304-405X(88)90021-9

[27] Fang Y, Xu D. The predictability of asset returns: an approach combining technical analysis and time series forecasts. International Journal of Forecasting. 2003;19(3):369–385. 10.1016/S0169-2070(02)00013-4

[28] Kercheval AN, Zhang Y. Modelling high-frequency limit order book dynamics with support vector machines. Quantitative Finance. 2015;15(8):1315–1329. 10.1080/14697688.2015.1032546

[29] Ntakaris A, Magris M, Kanniainen J, Gabbouj M, Iosifidis A. Benchmark dataset for mid-price forecasting of limit order book data with machine learning methods. Journal of Forecasting. 2018;37(8):852–866. 10.1002/for.2543

[30] Broomhead DS, Lowe D. Radial basis functions, multi-variable functional interpolation and adaptive networks. Royal Signals and Radar Establishment Malvern (United Kingdom); 1988.

[31] Iosifidis A, Tefas A, Pitas I. On the kernel extreme learning machine classifier. Pattern Recognition Letters. Pattern Recognition Letters. 2015;54:11–17. 10.1016/j.patrec.2014.12.003

[32] Richman JS, Moorman JR. Physiological time-series analysis using approximate entropy and sample entropy. American Journal of Physiology-Heart and Circulatory Physiology. 2000;278(6):H2039–H2049. 10.1152/ajpheart.2000.278.6.H2039 10843903

[33] Huang GB, Zhu QY, Siew CK. Extreme learning machine: theory and applications. Neurocomputing. 2006;70(1-3):489–501. 10.1016/j.neucom.2005.12.126

[34] Zhang K, Kwok JT, Parvin B. Prototype vector machine for large scale semi-supervised learning. Proceedings of the 26th Annual International Conference on Machine Learning. ACM; 2009. p. 1233–1240.

[35] Iosifidis A, Tefas A, Pitas I. Approximate kernel extreme learning machine for large scale data classification. Neurocomputing. 2017;219:210–220. 10.1016/j.neucom.2016.09.023

[36] Chua S. Sammy Chua’s Day Trade Your Way to Financial Freedom. John Wiley & Sons; 2006.

[37] Williams B. New trading dimensions: how to profit from chaos in stocks, bonds, and commodities. vol. 72, 1998 John Wiley & Sons; 1.

[38] Wilder JW Jr. The Relative Strength Index. Journal of Technical Analysis of Stocks and Commodities. 1986;4:343–346.

[39] Gregory-Williams J, Williams BM. TTrading chaos: maximize profits with proven technical techniques. vol. 172, 2012 John Wiley & Sons; 2012.

[40] Chande TS, Kroll S. The new technical trader. New York 1994;.

[41] Wilder JW. New concepts in technical trading systems Trend Research; 1978.

[42] Bollinger J. Bollinger on Bollinger bands. McGraw Hill Professional; 2001.

[43] Muranaka K. Ichimoku Charts. Technical Analysis of Stocks and Commodities–Magazine Edition. 2000;18(10):22–31.

[44] Naiman E. Small encyclopedia of trader. Moscow: Alpina Business Books. 2009;456.

[45] Elder A. Come into my trading room: A complete guide to trading. vol. 163, 2002 John Wiley & Sons; 2002.

[46] Ehlers JF. Rocket science for traders: digital signal processing applications. vol. 112, 2001 John Wiley & Sons; 2001.

[47] Rayome DL, Jain A, Konku D. Technical Analysis: Donchian Channels and the British Pound. IABE-Annual Conference; 2007. p. 302, 2007.

[48] Mulloy PG. Smoothing data with faster moving averages. Stocks & Commodities. 1994;12(1):11–19.

[49] Valcu D. Using The Heikin-Ashi Technique. Available from: Technical Analysis of Stocks and Commodities–Magazine Edition. 2004;22(2):16–29.

[50] Pagonidis AS. The IBS Effect: Mean Reversion in Equity ETFs; 2014. http://www.naaim.org/wp-content/uploads/2014/04/00V_Alexander_Pagonidis_The-IBS-Effect-Mean-Reversion-in-Equity-ETFs-1.pdf.

[51] Keltner CW. How to make money in commodities. Keltner Statistical Service; 1960.

[52] Aspray T. Individual stocks and MACD. Technical Analysis of Stocks & Commodities. 1989;7(2):56–61.

[53] Chande TS. Adapting moving averages to market volatility. Stock & Commodities. 1992;10:3.

[54] Tillson T. Better Moving Averages. Digital Filters. Monterrey; 1998.

[55] Blau W. Double smoothed-stochastics. Technical Analysis of Stocks and Commodities. 1991;9.

[56] Williams L. The Ultimate Oscillator. Technical Analysis of Stocks and Commodities. 1985;3(4):140–141.

[57] Kumaresan R. Identification of rational transfer function from frequency response sample. IEEE Transactions on Aerospace and Electronic Systems. 1990;26(6):925–934. 10.1109/7.62245

[58] Savitzky A, Golay MJ. Smoothing and differentiation of data by simplified least squares procedures. Analytical chemistry. 1964;36(8):1627–1639. 10.1021/ac60214a047

[59] Schafer RW. What Is a Savitzky-Golay Filter? IEEE Signal Processing Magazine. 2011;28(4):111–117. 10.1109/MSP.2011.941097

[60] Smith SW. The scientist and engineer’s guide to digital signal processing. California Technical Pub.; 1999.

[61] French CW. The Treynor capital asset pricing model. Journal of Investment Management. 2003;1(2):60–72.

[62] Box GE, Jenkins GM, Reinsel GC, Ljung GM. Time series analysis: forecasting and control. John Wiley & Sons; 2015.

[63] Eshel G. The Yule-Walker equations for the AR coefficients. Internet resource. 2003;2:68–73.

[64] Hamilton JD. Time series analysis. vol. 2 Princeton University Press Princeton; 1994.

[65] Engle RF, Granger CWJ. Co-Integration and Error Correction: Representation, Estimation, and Testing. Econometrica. 1987;55(2):251–276. 10.2307/1913236

